# Triazole-Based Functionalized Olygo(Arylene Ethynylene)s—Synthesis and Properties

**DOI:** 10.3390/molecules30234508

**Published:** 2025-11-22

**Authors:** Anastasia I. Govdi, Vasiliy V. Menchikov, Ilya E. Kolesnikov, Irina A. Balova

**Affiliations:** 1 Institute of Chemistry, Saint Petersburg State University (SPbU), Universitetskaya nab. 7/9, 199034 Saint Petersburg, Russia; a.govdi@spbu.ru (A.I.G.); st106275@student.spbu.ru (V.V.M.); 2 Center for Optical and Laser Materials Research, Saint Petersburg State University, Ulianovskaya 5, 198504 Saint Petersburg, Russia; ilya.kolesnikov@spbu.ru

**Keywords:** olygo(arylene ethynylene)s, 1,2,3-triazoles, azide–alkyne cycloaddition, Sonogashira cross-coupling, fluorescence, aggregation-induced emission enhancement

## Abstract

Oligo(arylene ethynylene)s (OAEs) containing 4,5-(diethynylaryl)-1,2,3-triazoles with 3(OMe) and NR_2_ substituents at the 5-position and bis-1,4-dialkoxybenzene moieties as spacers at the 4-position were obtained using the *retro*-Favorskii reaction as a key step. The most intense fluorescence was observed for OAEs with a trimethoxyphenyl substituent in THF solutions, with a quantum yield of up to 88%. Increasing the solvent polarity had minimal effect on the emission of trimethoxyphenyl substituted derivatives. A notable red shift in emission spectra was observed with increasing solvent polarity for OAEs **10a**,**g** containing para-dimethylaminophenyl group. Their emission spectra in aqueous organic solutions revealed that an increase in water fraction in THF/water mixtures led to a bathochromic shift in emission spectra maxima accompanied by a hypochromic effect. An increase in intensity was observed in aqueous acetonitrile and DMSO. The maximum intensity was observed in DMSO solutions containing 30% water, which is attributed to aggregate-induced emission enhancement. Dynamic light scattering data also confirmed the formation of nanoscale aggregates in aqueous organic mixtures.

## 1. Introduction

Conjugated organic oligomers and polymers have emerged as one of the most dynamic fields of scientific research over the past two to three decades [1,2,3,4,5]. These materials have demonstrated broad applicability in a variety of areas, including organic electroluminescent devices (OLEDs) [6], supramolecular chemistry [7,8,9], organic photovoltaics [10,11], biosensors [12,13,14], and chemical sensors [15,16,17,18,19,20].

However, a number of challenges [21] associated with polymeric materials, such as difficulties in purification, determination of their exact structure, and broad molecular weight distribution, have spurred intense research into well-defined small molecules or oligomers [22,23,24]. Oligo(arylene ethynylene)s (OAEs) with defined length and various anchor groups offer several advantages due to their rigidity, rod-like structure, ease of purification, and precise control over functional properties. Furthermore, their properties can be precisely tailored by varying the terminal substituents and the length of the oligomeric chain, as well as by the modification of the arylene core electronic nature and the introduction of pendant groups [25,26]. This feature also makes them highly sensitive in molecular recognition, as the entire polymer chain exhibits an amplified fluorescence quenching response. In oligo(arylene ethynylene)s, the specificity towards various analytes can be fine-tuned by modifying the rigid conjugated segments onto the main chain or by introducing functional substituents on the side chains, without disrupting the conjugation pathway.

For potential biological applications as fluorescent probes, a significant challenge is to shift emission to the red region. To achieve this bathochromic wavelength shift, a common approach is to increase in conjugation of fluorophores by introducing electron-donating and electron-withdrawing substituents into their structural fragments. Fluorescent molecules of the D-π-A type, obtained in this way, are of particular interest. In molecules of this class, the excited state is usually strongly polar compared to the ground state due to intramolecular charge transfer (ITC) from the donor group to the acceptor group [27].

Recently, we reported that 4,5-diethynyltriazoles derivatives exhibit promising luminescent properties [28,29]. Considering the electron-deficient nature of the 1,2,3-triazole core, we decided to use it as a structural element of the main chain OAEs, while simultaneously introducing aryl fragments with electron-donating substituents through triple bonds as linear linkers, incising the conjugation of the chain. In addition, the substituents on the N-1 position of the triazole ring allow for the introduction and variation in functional groups in the side chain, ensuring efficient molecular functionalization. Therefore, the development of synthetic routes to fully conjugated polymers where 1,2,3-triazole moieties could act as molecular recognition sites, followed by their structural modification, is a relevant research goal. Achieving this would enable the creation of new fluorescent materials with tenable sensing characteristics for bioimaging applications.

This work describes the synthesis of novel 4,5-diethynyl-1,2,3-triazole derivatives with enhanced π-conjugated chains and investigates the relationship between their molecular structure and photophysical properties.

## 2. Results and Discussion

### 2.1. Synthesis of 1,2,3-Triazole-Containing Oligo(Arylene Ethynylene)s (Trz-OAEs)

In our previous study [30], we demonstrated, that 1-iodobuta-1,3-diynes are useful starting materials for the synthesis of 5-iodo-4-ethylnyltriazoles in the copper-catalyzed azide-alkyne cycloaddition (CuAAC). In turn, these compounds are valuable because their subsequent modification via palladium-catalyzed cross-coupling reactions allows access to structures with unique properties, such as fluorescent molecules and systems based on heterocyclic enediyne analogs [31].

**Figure 1:**
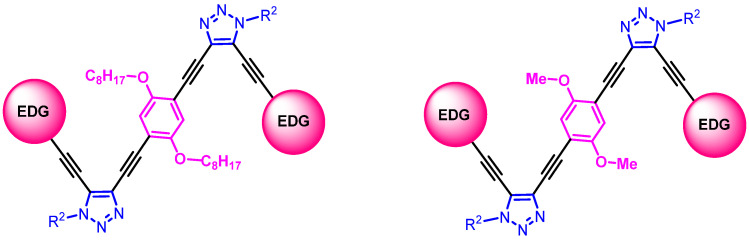
Structures of triazole-based oligo(arylene ethynylene)s (Trz-OAEs) **10**.

The Sonogashira cross-coupling is a useful tool for the synthesis of various oligomeric structures, in particular molecules consisting of alternating aromatic fragments and acetylene units as oligo(arylene ethynylene)s. The key structures for this purpose are diethynyl-substituted aromatic or heterocyclic rings. To study the relationship between structure and photophysical properties, we decided to obtain triazole-based oligo(arylene ethynylene)s with different donor substituents at the 5-position and bis-1,4-dialkoxybenzenes moieties as spacers at the 4-th position of the triazole ring (Figure 1).

**Scheme 1:**
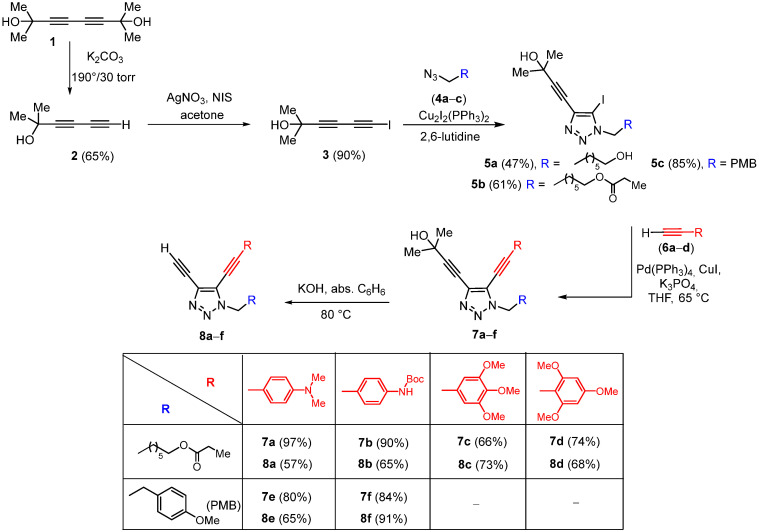
Synthesis of 4,5-diethynyl-substituted triazoles **8a**–**f**.

We used a five-step procedure that includes three sequential *retro*-Favorskii reactions [32] to obtain 4,5-diethynyltriazoles **8** with a terminal alkyne at the 4-position of the cycle. An advantage of this approach is the accessibility of all the starting reagents. 6-Iodo-2-methylhexa-3,5-diyn-2-ol **3** was synthesized in high yield according to our previously developed procedures [30]. The sequence also included the copper-catalyzed 1,3-dipolar cycloaddition of azides **4a**–**c** to 1-iodobuta-1,3-diyne (CuAAC), and the Sonogashira cross-coupling of 4-ethynyl-5-iodotriazoles **5** with arylacetylenes **6** resulted in 4,5-diethynyl-substituted triazoles **8a**–**f** (Scheme 1).

Initial experiments revealed that the reaction of iododiacetylene **3** with azidohexanol **4a** using the catalytic system [Cu_2_I_2_(PPh_3_)_2_]_2_/2,6-lutidine proceeded with low conversion of the starting iododiyne. After 30 h, the yield of the 5-iodo-1,2,3-triazole **5a** did not exceed 50%, even when the catalyst loading reached 20 mol%.

We hypothesized that the presence of two hydroxyl groups in the substrate leads to catalyst deactivation. Therefore, we synthesized azide with an acylated hydroxyl group (**4b**). The CuAAC between 1-iododiacetylenic alcohol **3** and azide **4b** still proceeded rather slowly. In order to achieve complete conversion of iododiacetylene alcohol **3**, it was necessary to heat the reaction mixture at 40 °C for 18 h in the presence of 10 mol% catalyst, which significantly increased the yield of the resulting triazole **5b**.

We selected 4-ethynyl-5-iodo-1,2,3-triazoles **5b**,**c** for further modification with aryl acetylenes **6a**–**d** bearing electron-donating substituents (EDG) under the Sonogashira reaction conditions. This approach allows the synthesis of acyclic enediyne systems conjugated with the triazole ring and bearing various functional groups on both ethynyl fragments. The Sonogashira reaction of 5-iodotriazoles **5b**,**c** with alkynes **6a**–**d** was carried out using potassium phosphate as a base and Pd(PPh_3_)_4_ as a catalyst in THF at 65 °C. This procedure afforded 4,5-diethynyltriazoles **7a**–**f** in good yields. Then the acetone protecting group was removed from triazoles **7a**–**f** using a classical *retro*-Favorskii reaction with calcined potassium hydroxide upon heating in anhydrous benzene. The desired terminal alkynes **8a**–**f** were obtained in good yields (Scheme 1).

**Scheme 2:**
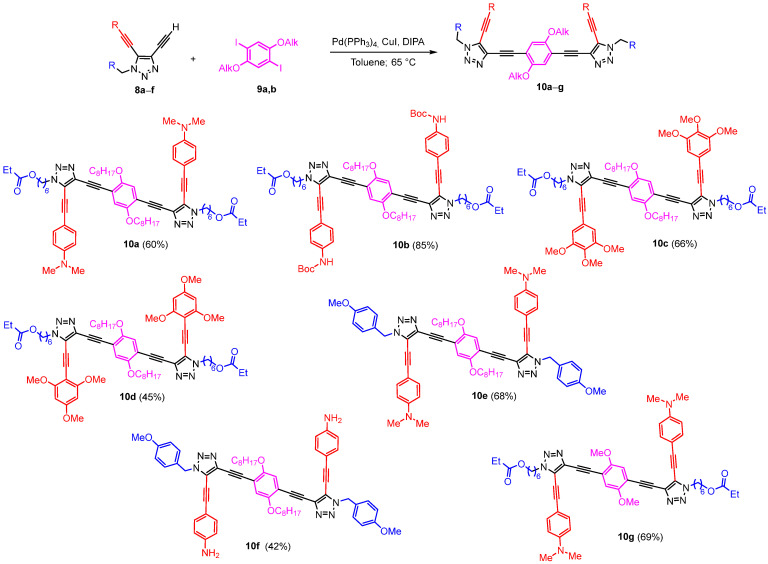
Synthesis of Trz-OAEs **10a**–**g**.

The use of the Pd-catalyzed Sonogashira cross-coupling in a step-growth polycondensation has proven to be an efficient synthetic route to OAEs [33]. Reactions of 4,5-diethynyltriazoles **8a**–**f** with terminal triple bond and *O*-alkylated 2,5-diiodohydroquinone **9a**,**b**, acting as a spacer between the diethylnyltriazole units, were performed under standard conditions, using Pd(PPh_3_)_4_/CuI catalytic system and DIPA as a base. The reaction was carried out by heating in toluene, obtaining products **10a**–**g** in yields of 45–85% (Scheme 2). Compound **10f** with a primary amino group was obtained from a *N*-Boc-protected precursor **10f’** (see Materials and Methods) by removing the Boc group with trifluoroacetic acid (TFA). We then turned to the study of the optical properties of compounds **10a**,**c**–**g**.

### 2.2. Photophysical Properties of 1,2,3-Triazole-Containing Oligo(Arylene Ethylene)s

**Figure 2:**
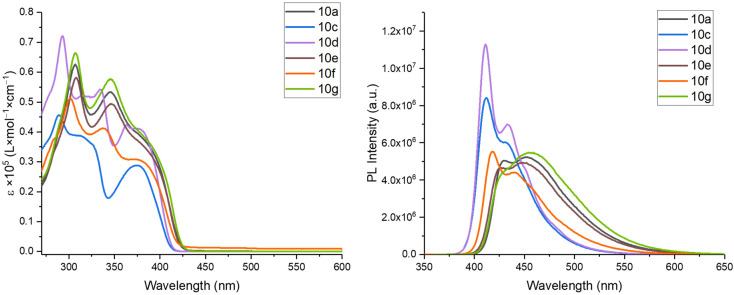
Absorption (left) and emission (right) spectra of compounds **10a,c**–**g** in THF, C_M_ = 10^−5^ M.

It was found that compounds **10a**,**c**–**g** in THF solutions exhibit absorption bands in the 260–300 nm region, corresponding to a π-π* electronic transition, along with less intense absorption maxima in the 290–320 nm region, characteristic of an n-π* transition. Additionally, extra absorption bands appear in the 340–350 nm range (for **10a**,**e**,**g**) and the 365–400 nm range (for **10c**,**d**), which are also attributed to π-π* electronic transitions (Figure 2 and Table 1).

Analysis of the emission spectra recorded for solutions of Trz-OAEs **10a**,**c**–**g** in THF revealed that compounds **10c** and **10d**, which contain trimethoxyphenyl substituents, exhibit the most intense fluorescence with the highest quantum yield and with a small Stokes shift compared to OAEs with amino groups in this series (Table 1). The emission maxima for **10c**,**d** lie in the 410 nm region. Replacing the trimethoxyphenyl substituents with a dimethylaminophenyl group in compounds **10a**,**e**, and **g** results in a bathochromic (red) shift in the emission maxima to 445–455 nm, accompanied by a 2.2-fold decrease in the photoluminescence quantum yield for **10a** (**Φ_F_** = 32%) compared to **10c** (**Φ_F_** = 88%). Notably, the compound **10f** containing a primary amine group exhibits the smallest Stokes shift among its dimethylamino analogs **10a**,**e**,**g**, similar to trimethoxyphenyl substituted analogs.

### 2.3. Solvatochromic Properties

**Figure 3:**
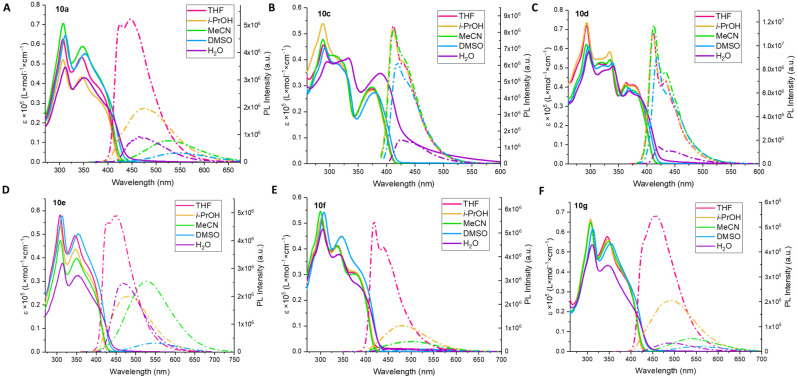
Absorption (solid line) and Emission spectra (dashed line) of Trz-OAEs **10a** (**A**), **10c** (**B**), **10d** (**C**), **10e** (**D**), **10f** (**E**), and **10g** (**F**) in various solvents.

An important task for the design of fluorescent chemosensors that can convert binding with analytes into a measurable signal through changes in fluorescence parameters is to investigate the effect of solvation in various solvents on the brightness of chromophores. To investigate the solvatochromism of the synthesized Trz-OAEs, their absorption and emission spectra were recorded in various solvents (tetrahydrofuran, isopropanol, acetonitrile, DMSO, water) at a concentration of 10^−5^ M (Figure 3). The solvents were selected based on their solvatochromic parameter (π*) [34], which characterizes the solvent’s ability to stabilize a charge or dipole through dielectric effects. Considering the potential applications of the obtained OAEs for biochemical purposes, protic and aprotic solvents that are miscible with water were selected. A clear difference in the emission spectra of OAEs **10a**,**c**–**g** with two types of donor substituents was found depending on the nature of the solvent. For compounds with trimethoxyphenyl substituents **10c**,**d**, increasing solvent polarity caused only a weak bathochromic shift in maxima (no more than 12 nm relative to the maximum in THF). No noticeable decrease in quantum yields with increasing the solvent polarity (excluding water) is observed; moreover, for **10d** in acetonitrile, the quantum yield increased (**Φ_F_** = 78%). This behavior indicates that compounds **10c**,**d** with trimethoxyphenyl substituents, which are strong donors but less susceptible to specific interactions with solvent molecules than those containing amino groups, have a limited change in the dipole moment upon molecular excitation. A small value of the Stokes shift typically indicates a weak solvent reorganization and little vibrational relaxation regardless of solvent polarity. The exception was aqueous medium, where aggregation effects likely predominate, leading to an increase in the proportion of nonradiative relaxation, for example, through solvent-assisted electronic-to-vibrational FRET and hydrogen bonding-induced quenching, which are common processes that affect the efficiency of fluorescence [35].

In contrast, compounds with para-(dimethylamino)phenyl substituents (**10a**,**e**,**g**) exhibit a significant increase in Stokes shifts to 200 nm upon moving toward polar solvents, accompanied by a noticeable decrease in fluorescence intensity, and absolute quantum yields decreased from 56 to 7%. Amino groups with readily polarized lone pairs of electrons are common components of organic dyes due to their strong electron-donating properties, which result in interesting electronic and geometric properties, promoting intramolecular charge transfer (ICT) in an electronically excited state. The introduction of an amino group also significantly affects the fluorescence quantum yield [36]. This property is explained by various mechanisms, including twisted intramolecular charge transfer (TICT), which introduces additional pathways for non-radiative energy dissipation.

Observed changes in the emission spectra of OAEs **10a**,**e**,**g** indicate a significant increase in the excited-state dipole moment and the formation of a highly polarized and possibly non-emissive twisted intramolecular charge-transfer (TICT) state. TICT, which involves the formation of a charge-transfer (CT) state with a perpendicular conformation through intramolecular rotation around a chemical bond, has been commonly proposed and observed for amines that can rotate around the amine-based chain bond [35,36]. We have recently reported that ethynyltriazole molecules are not perfectly planar; the dimethylaminophenyl group is rotated by approximately 79 degrees relative to the plane of the triazole core [28]. In compounds **10a**,**e**–**g**, the amino-phenyl group can also be rotated with respect to the triazole ring. This non-planar conformation of the ground state may indicate a tendency of the molecule to undergo significant rotation in the rotational angle after photostimulation. In polar solvents, this relaxation can be stabilized, resulting in the formation of a fully orthogonal TICT (twisted intramolecular charge transfer) state, which could explain the observed abrupt fluorescence quenching.

Remarkably, compound **10f** (bearing a free NH_2_ group) exhibits both positive and negative solvatochromism simultaneously. When moving from THF to acetonitrile, a bathochromic shift in the fluorescence maximum is observed, which lies in a blue-green region (481–527 nm). However, a further increase in solvent polarity, such as in water, leads to a hypsochromic shift (468 nm). The sharp decrease in photoluminescence intensity of this compound in polar solvents can be attributed to a combination of factors.

The sharp decrease in the photoluminescence intensity of compound **10f** with increasing solvent polarity is usually attributed to photoinduced electron transfer (PET). The high-energy lone pair on the nitrogen atom of the NH_2_ group transfers an electron to the fluorophore core, effectively suppressing fluorescence. In contrast, the NMe_2_ group in other compounds is sterically shielded, and its corresponding radical cation is more stable, making PET less favorable.

To confirm our hypothesis about the possible mechanism for luminescence quenching in PET-based compounds, we recorded absorption and emission spectra of compound **10f** in THF and acetonitrile solutions in the presence of trifluoroacetic acid (Figure S1, Supplementary Information). Expected change in the absorption spectra, adding various ratios of TFA to the amine **10f** solution, was not observed. The emission intensity decreased, but only slightly, even with a twofold excess of TFA. Protonation of the amino group should have resulted in either a sharp increase or a sharp decrease in emission intensity, but we observed neither effect. This behavior of compound **10f** indicates the absence of photoinduced electron transfer. Therefore, another mechanism for fluorescence quenching in polar protic solvents can be hypothesized, involving possible solvent–solute interactions via the formation of intermolecular hydrogen bonds and aggregation effects.

**Figure 4:**
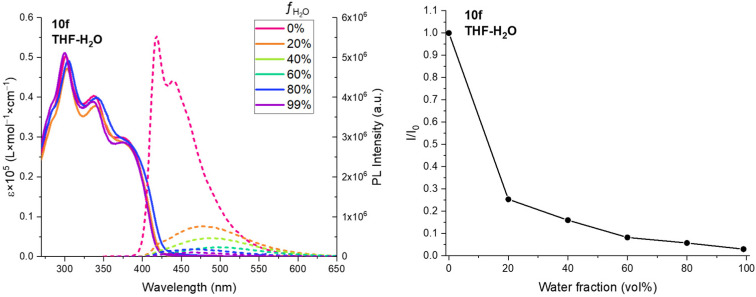
Absorption (solid line) and Emission spectra (dashed line) of Trz-OAEs **10f** in THF/Water and Plots of (I/I0) values of the compositions of the aqueous mixtures.

The primary amino group is particularly prone to forming hydrogen bonds with both protic (e.g., propanol, water) and aprotic (e.g., DMSO, CH_3_CN) solvents. These hydrogen bonds stabilize the ground state and alter energy levels, facilitating nonradiative relaxation and contributing to quenching of fluorescence and an unusual blue shift in highly polar media. Spectra recorded for a solution of **10f** in THF with varying water concentrations (Figure 4) support the idea that non-radiative relaxation, resulting in the formation of hydrogen bonds, is the main mechanism for quenching the photoluminescence of compound **10f**. The addition of 20% water causes a dramatic sevenfold decrease in emission intensity, while a further increase in the water concentration leads to almost complete quenching of luminescence.

For compounds **10a** and **10e**, a decrease in intensity is also observed but occurs more gradually, consistent with a different quenching mechanism.

To quantitatively assess the observed fluorescence, the absolute quantum yields (**Φ_F_**) for compounds **10a**–**g** were measured in THF, DMSO, MeCN, and *i*-PrOH solutions. The quantum yield data are presented in Table 2.

The luminescence kinetic curves of **10c**, **10d**, and **10g** in various solvents are presented in SI with the corresponding data approximations. Most of the experimental decay curves can be fit by a single-exponential function, but for some samples (particularly water and DMSO), a bi-exponential approximation is necessary. This is because of the higher likelihood of non-radiative processes in these instances. The radiative (k_r_) and nonradiative (k_nr_) decay rates for compounds **10c**, **10d**, and **10g** were calculated across a range of solvents and are summarized in Table S1 (Supplementary Information, S5). The findings of the study demonstrated that **10c** exhibited the highest radiative decay rates, while the lowest values were determined for **10g**. Moreover, **10c** exhibits the lowest non-radiative decay rates in comparison to other molecules. It has been demonstrated that water solution compounds exhibit the lowest radiative decay rates in comparison to all other solvents.

**Figure 5:**
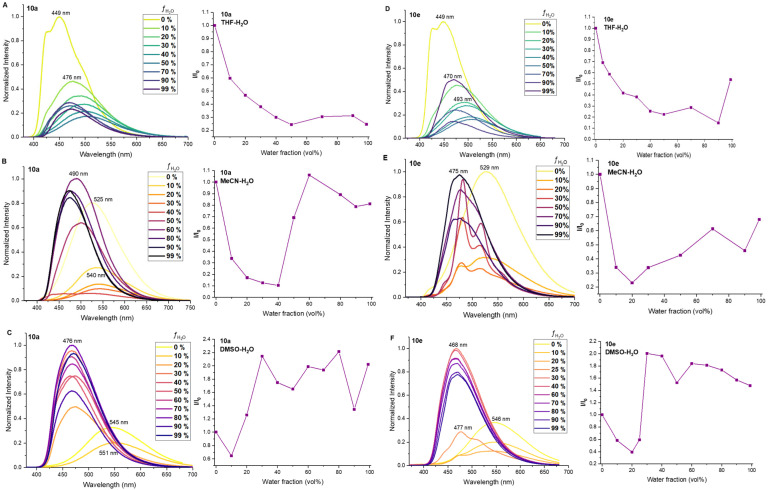
(**A**–**C**) Fluorescence response of probe **10a** upon increasing concentration of water fraction in THF (**A**), MeCN (**B**), DMSO (**C**) (λex 352 nm), and Plots of (I/I_0_) values the compositions of the aqueous mixtures; (**D**–**F**) Fluorescence response of probe **10e** upon increasing concentration of water fraction in THF (**D**), MeCN (**E**), DMSO (**F**) (λex 352 nm), and Plots of (I/I_0_) values the compositions of the aqueous mixtures, C_M_ = 10^−5^ M; I_0_ = fluorescence intensity in the absence of water.

Then we studied emission spectra of the synthesized compounds **10a** and **10e** in different solvents—tetrahydrofuran (THF), acetonitrile (MeCN), and dimethyl sulfoxide (DMSO), with varying concentrations of water. OAEs bearing a dimethylaminogroup in the THF/water mixture, an increase in the water fraction led to a gradual bathochromic shift in the emission spectra for both compounds, accompanied by a hypsochromic effect. When the content exceeded 50%, only a slight hypsochromic shift and a minor increase in emission intensity were observed (Figure 5A,D).

In the MeCN/water mixture, the behavior of the compounds was more complex. For compound **10a**, the initial addition of water caused a bathochromic shift, along with a decrease in luminescence intensity. However, after reaching 40% vol. of water, the emission intensity began to increase (hyperchromic effect), reaching its maximum at 60% water. This rise in intensity was accompanied by a hypsochromic shift in the emission peak to 490 nm (Figure 5B). A similar trend was observed for compound **10e**. Starting from a 30% volume of water, two emission maxima appeared, which indicated probably the existence of additional local excited states; the emission intensity gradually increased with the rising water fraction. When the water content reached 50%, only the short-wavelength maximum at 475 nm remained, and its position did not change thereafter. Interestingly, when the water fraction increased from 70% to 90%, a hypsochromic effect was observed, and the second maximum emission disappeared. However, a further increase in water content to 99% resulted in a notable hyperchromic effect, where the emission intensity became comparable to that of pure acetonitrile (Figure 5E).

In the DMSO/water system, both compounds **10a** and **10e** exhibited similar behavior. Without water, they had relatively weak fluorescence intensity. However, when water was added, there was a significant hypsochromic shift in the emission wavelength from 546 nm to 471 nm, accompanied by a substantial increase in emission intensity. The maximum increase in intensity was observed in DMSO solutions with the addition of 30% water. As the concentration of water increased further, the emission wavelength remained relatively unchanged, and the intensity varied only slightly. A clear AIEE effect, where the emission intensity at high water content exceeds that in pure solvent, can be unambiguously observed in DMSO/water mixtures for compounds **10a** and **10e**. However, in MeCN/water systems, even at its maximum, the emission intensity does not exceed the initial value in pure MeCN. This non-monotonic behavior suggests that multiple competing processes are involved. The reason for this behavior is likely multifaceted and may involve factors such as a change in molecular conformation of OAEs, which contain parts with different polarity upon aggregation, the formation of H-aggregates, or intramolecular charge transfer (ICT), which collectively contribute to the observed blue shift and fluorescence enhancement.

To confirm the aggregate formation, we performed dynamic light scattering (DLS) measurements on aqueous solutions and in pure DMSO. For compound **10a** in pure DMSO, no significant aggregation was observed, with particle sizes ranging from 1 to 14 nm. However, in a solution with 80% water, the particle size increased significantly to a range of 103–109 nm. A different behavior was observed for compound **10e**. Due to the absence of long octyl substituents, large aggregates formed even in pure DMSO, with a broad range of sizes from 202 to 2434 nm. This extensive aggregation results in low fluorescence intensity. Interestingly, when 30% water was added, the aggregate decreased, and a more uniform particle distribution (250–450 nm) was observed, which correlates with an increase in emission intensity.

Thus, our findings show that the observed non-monotonic change in fluorescence intensity (initial decrease followed by an increase) is directly related to aggregate formation at different water fractions. In this DMSO/H_2_O system, the aggregates exhibit a dual characteristic; they can either quench or enhance the fluorescence intensity at specific water percentages. The above results conclusively prove that the synthesized Trz-OAEs exhibit distinct aggregation-induced emission enhancement (AIEE) properties.

**Scheme 3:**
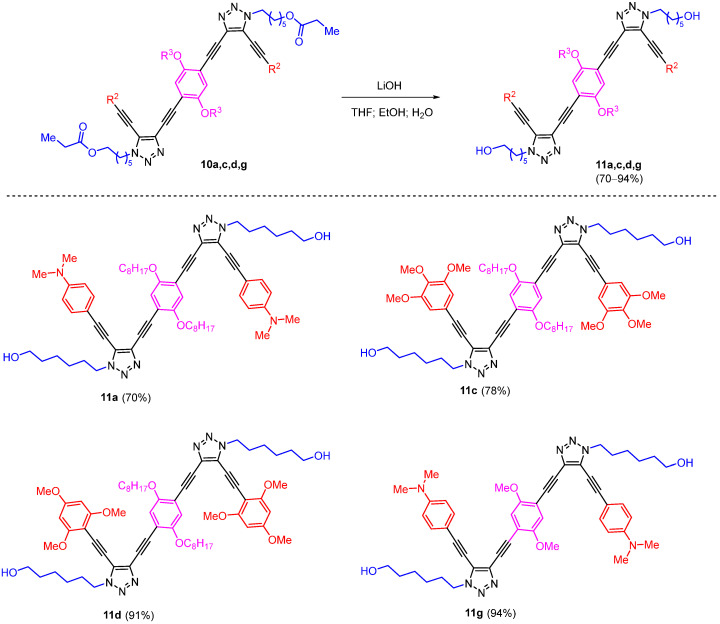
Hydrolysis of esters **10a**,**c**,**d**,**g** to the corresponding alcohols **11a**,**c**,**d**,**g**.

For compounds **10a**,**c**,**d**,**g**, with an ester group, hydrolysis was carried out using a lithium hydroxide solution in a THF/water mixture. However, for some of these compounds, the addition of ethanol was necessary to improve the solubility of the starting materials. As a result, triazoles with free hydroxyl groups (**11a**,**c**,**d**,**g**) were obtained in high yields (Scheme 3).

The optical properties of the synthesized compounds were also investigated. The trends in the absorption and emission spectra for compounds **11a**,**c**,**d**,**g** mirror those of their precursors, **10a**,**c**,**d**,**g**.

**Figure 6:**
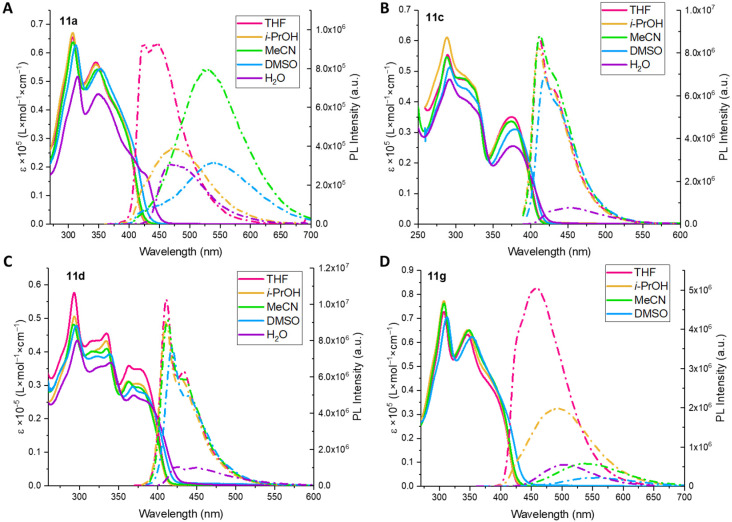
UV-Vis absorption (solid line) and photoluminescence (PL, dashed line) emission spectra of compounds **11a** (**A**) **, 11c** (**B**)**, 11d** (**C**) and **11g** (**D**) in solvents of different polarity. (C = 10^−5^ M).

Compounds **11a** and **11g**, bearing a phenyldimethylamino group, exhibit the maximum emission intensity in tetrahydrofuran (THF), with emission maxima at 444 and 457 nm, respectively. Their photoluminescence quantum yields are also the highest in THF, reaching 48% and 39% (Table 3). The transition from THF to more polar solvents (*i*-PrOH, MeCN, DMSO, H_2_O) leads to a significant decrease in emission intensity. This is accompanied by a small bathochromic shift of 28–35 nm in *i*-PrOH and water, whereas a more substantial bathochromic shift of 87 and 97 nm is observed in acetonitrile and DMSO, respectively (Figure 6A,D). The quantum yields also decrease correspondingly. This behavior suggests that the molecule’s excited state is more polar than its ground state; consequently, in more polar solvents, the excited state is stabilized more effectively than the ground state.

Compound **11a** exhibits a notable exception in its solvatochromic behavior; its emission in acetonitrile is significantly red-shifted to 531 nm (Stokes shift = 184 nm) compared to its emission in THF. Despite this substantial bathochromic shift, which often coincides with quenching, the reduction in emission intensity is only moderate (Figure 6D).

Replacing phenyldimethylamino with the trimethoxyphenyl group in compounds **11c** and **11d** also results in a significant increase in emission intensity. This is reflected in their high photoluminescence quantum yields of 70–75% in DMSO. Furthermore, solvent polarity has a negligible effect on the position of the maxima, the intensity, or the shape of the absorption and emission spectra for these two compounds. Their primary emission maxima lie within the violet region around 410–450 nm (Table 3 and Figure 6 B,C).

## 3. Materials and Methods

### 3.1. General Information

All solvents and reagents were obtained from commercial suppliers and used as received, unless otherwise specified. Solvents for reactions were dried according to standard laboratory methods. The catalyst tetrakis(triphenylphosphine)palladium(0) (Pd(PPh_3_)_4_) was sourced from Sigma-Aldrich (München, Germany). The starting materials, including the dimeric copper(I) complex [Cu_2_I_2_(PPh_2_)]_2_ [37], 1-iodobuta-1,3-diyne **3**, and organic azides **4a**–**c** [30] were prepared according to the previously reported literature procedures. Reaction mixtures were concentrated under reduced pressure using a rotary evaporator with a water bath temperature set at 35 °C. Reaction progress was monitored by thin-layer chromatography (TLC) on pre-coated silica gel plates (Silica gel 60 F_254_, Merck, Darmstadt, Germany) with visualization under UV light (λ = 254 nm). NMR spectroscopic data for all new compounds are provided in the Supplementary Materials. ^1^H and ^13^C NMR spectra were recorded in CDCl_3_ at 25 °C on a Bruker Avance spectrometer (Bruker, Billerica, MA, USA). Chemical shifts (δ) are reported in parts per million (ppm). The ^1^H NMR data are presented as follows: chemical shift, multiplicity (s = singlet, d = doublet, t = triplet, q = quartet, m = multiplet), coupling constant (J) in Hertz (Hz), and integrated proton count. High-Resolution Mass Spectrometry (HRMS) was performed on a Bruker microTOF instrument using electrospray ionization (ESI) (Billerica, MA, USA )in positive ion mode. UV-Vis spectra were recorded at room temperature on a Shimadzu UV-1800 spectrophotometer (Shimadzu, Kyoto, Japan). Fluorescence spectra were measured at room temperature on a Horiba Scientific FluoroMax-4 spectrofluorometer (Horiba Scientific, Glasgow, Scotland).

Fluorescence decay curves were measured on a modular Fluorolog-3 spectrofluorometer (Horiba Jobin Yvon, Tokyo, Japan) with a light-emitting diode as an excitation source (emission wavelength 340 nm, pulse duration 1.2 ns, repetition rate 1 MHz). Fluorescence lifetimes were obtained using the TCSPC technique. The photoluminescence quantum yields were determined by an absolute method using a Fluorolog-3 spectrofluorometer equipped with an integrating sphere Quanta-phi (6 inches).

### 3.2. Synthetic Methods and Analytic Data of Compounds

#### 3.2.1. General Procure for the CuAAC

To an azide (1.00 equiv.) in a vial were consistently added 1-iodobuta-1,3-diyne (1.00 equiv.), [Cu_2_I_2_(PPh_2_)]_2_ (1.5–5 mol%) and 2,6-lutidine (4–8 mol%). The thick resulting mixture was vigorously stirred overnight at room temperature or 40 °C (TLC control). Then the reaction mixture was diluted with CH_2_Cl_2,_ and the resulting solution was washed with a saturated aqueous solution of NH_4_Cl. The mixture was extracted, and the combined organic layers were dried over Na_2_SO_4_, filtered, and concentrated. The residue was purified by column chromatography on silica gel.

6-(4-(3-Hydroxy-3-methylbut-1-yn-1-yl)-5-iodo-1H-1,2,3-triazol-1-yl)hexan-1-ol (**5a**) was prepared in accordance with the general procedure from 6-iodo-2-methylhexa-3,5-diyn-2-ol **3** (100.0 mg, 0.43 mmol), 6-azidohexan-1-ol **4a** (85.6.0 mg, 0.60 mmol), [Cu_2_I_2_(PPh_2_)]_2_ (64.2 mg, 0.086 mmol, 20 mol%) and 2,6-lutidine (3.7 mg, 0.034 mmol, 8 mol%). The reaction mixture was stirred for 30 h at 40 °C. The crude product was purified by column chromatography (eluent:hexane/acetone = 2:1) to afford brown viscous oil (76 mg, 47%). ^1^H NMR (400 MHz, CDCl_3_) δ 4.37 (t, *J* = 7.2 Hz, 2H), 3.17 (t, *J* = 6.9 Hz, 2H), 2.17 (s, 1H), 1.92 (p, *J* = 7.3 Hz, 2H), 1.81 (p, *J* = 7.0 Hz, 2H), 1.65 (s, 6H), 1.50–1.40 (m, 2H), 1.40–1.30 (m, 1H).

6-(4-(3-Hydroxy-3-methylbut-1-yn-1-yl)-5-iodo-1*H*-1,2,3-triazol-1-yl)hexyl propionate (**5b**) was prepared in accordance with the general procedure from 6-iodo-2-methylhexa-3,5-diyn-2-ol **3** (300 mg, 1.28 mmol), 6-azidohexyl propionate **4b** (383 mg, 1.79 mmol), [Cu_2_I_2_(PPh_2_)]_2_ (96.3 mg, 0.064 mmol, 5 mol%) and 2,6-lutidine (11 mg, 0.10 mmol, 8 mol%). The reaction mixture was stirred overnight at 40 °C. The crude product was purified by column chromatography (eluent:hexane/acetone = 3:1) to afford a brown, viscous oil (350 mg, 61%). ^1^H NMR (CDCl_3_, 400 MHz) δ 4.35 (t, *J* = 7.2 Hz, 2H), 4.05 (t, *J* = 6.6 Hz, 2H), 2.36–2.26 (m, 2H), 1.97–1.84 (m, 2H), 1.45–1.30 (m, 4H), 1.13 (t, *J* = 7.6 Hz, 3H). ^13^C NMR (CDCl_3_, 101 MHz) 174.7, 137.6, 99.8, 84.1, 65.7, 64.2, 51.2, 31.3, 29.7, 28.5, 27.7, 26.1, 25.5, 9.3. HRMS ESI: [M + Na]^+^ calcd. for C_16_H_24_IN_3_NaO_3_^+^: 456.0755; found: 456.0759.

4-(5-Iodo-1-(4-methoxybenzyl)-1*H*-1,2,3-triazol-4-yl)-2-methylbut-3-yn-2-ol (**5c**) was prepared in accordance with the general procedure from 6-iodo to 2-methylhexa-3,5-diyn-2-ol **3** (200 mg, 0.85 mmol), [Cu_2_I_2_(PPh_3_)_2_]_2_ (32.1 mg, 0.021 mmol, 5 mol%), 2,6-lutidine (3.66 mg, 0.034 mmol, 4 mol%) and 1-(azidomethyl)-4-methoxybenzene **4c** (153 mg, 0.94 mmol). The reaction mixture was stirred overnight at RT. The crude product was purified by column chromatography (eluent:hexane/EtOAc = 3:1) to afford a white solid (290 mg, 85%). ^1^H NMR (400 MHz, CDCl_3_) δ 7.25–7.19 (m, 2H), 6.90–6.81 (m, 2H), 5.50 (s, 2H), 3.78 (s, 3H), 1.62 (s, 6H). ^13^C NMR (101 MHz, CDCl_3_) δ 160.0, 138.1, 129.6, 126.0, 114.4, 99.9, 84.1, 71.8, 65.7, 55.5, 54.4, 31.3. HSMS ESI: [M + H]^+^ calcd for C_15_H_16_IN_3_O_2_^+^ 398.0360; found: 398.0367.

#### 3.2.2. General Procedure for the Sonogashira Cross-Coupling

A mixture of 5-iodo-1*H*-1,2,3-triazole **5b** or **5c** (1.0 equiv.), Pd(PPh_3_)_4_ (5 mol%), CuI (10 mol%), and K_3_PO_4_ (1.1 equiv.) were placed in a vial. The vial was sealed, evacuated, and refilled with argon (three cycles). Tetrahydrofuran (1 mL) was added, and the mixture was stirred at room temperature for 10 min. The respective alkyne **6a**–**d** (1.2 equiv.) was then introduced, and the reaction vial was immersed in a pre-heated aluminum block at 65 °C. The mixture was stirred for 5–6 h, with the reaction progress monitored by TLC. After cooling to ambient temperature, the mixture was filtered through a short pad of silica gel, eluting with CH_2_Cl_2_ (3 × 10 mL). The combined filtrates were concentrated under reduced pressure, and the crude residue was purified by column chromatography on silica gel.

6-(5-((4-(Dimethylamino)phenyl)ethynyl)-4-(3-hydroxy-3-methylbut-1-yn-1-yl)-1*H*-1,2,3-triazol-1-yl)hexyl propionate (**7a**) was prepared in accordance with the general procedure from 5-iodo-1*H*-1,2,3-triazole **5b** (80 mg, 0.19 mmol), 4-ethynyl-*N,N*-dimethylaniline **6a** (29.5 mg, 0.2 mmol), Pd(PPh_3_)_4_ (10.7 mg, 0.0092 mmol), CuI (3.5 mg, 0.019 mmol) and K_3_PO_4_ (43 mg, 0.2 mmol) with a reaction time of 6 h. The crude product was purified by column chromatography (eluent: hexane/acetone = 2:1) to afford a brown, viscous oil (81 mg, 97%). ^1^H NMR (400 MHz, CDCl_3_) δ 7.46–7.36 (m, 2H, Ar), 6.72–6.64 (m, 2H, Ar), 4.41 (t, *J* = 7.0 Hz, 2H, CH_2_), 4.05 (t, *J* = 6.5 Hz, 2H, CH_2_), 3.04 (s, 6H, NMe_2_), 2.31 (q, *J* = 7.6 Hz, 2H, CH_2_), 2.04–1.94 (m, 2H, CH_2_), 1.66 (s, 6H, C(CH_3_)_2_), 1.65–1.58 (m, 2H, CH_2_), 1.45–1.35 (m, 4H, 2CH_2_), 1.13 (t, *J* = 7.6 Hz, 3H, CH_3_). ^13^C NMR (101 MHz, CDCl_3_) δ 174.7, 151.1, 133.1, 132.3, 124.9, 111.8, 107.3, 104.8, 99.7, 71.8, 71.5, 65.7, 64.2, 49.3, 40.2, 31.3, 29.6, 28.6, 27.7, 26.2, 25.5, 9.2; HSMS ESI: [M + H]^+^ calc for C_26_H_34_N_4_O_3_^+^ 451.2704; found: 451.2695.

6-(5-((4-((*tert*-Butoxycarbonyl)amino)phenyl)ethynyl)-4-(3-hydroxy-3-methylbut-1-yn-1-yl)-1*H*-1,2,3-triazol-1-yl)hexyl propionate (**7b**) was prepared in accordance with the general procedure from 5-iodo-1*H*-1,2,3-triazole **5b** (100 mg, 0.23 mmol), alkyne **6b** (55.2 mg, 0.25 mmol), Pd(PPh_3_)_4_ (13.3 mg, 0.0115 mmol), CuI (4.4 mg, 0.023 mmol) and K_3_PO_4_ (53.9 mg, 0.25 mmol) with a reaction time of 6 h. The crude product was purified by column chromatography (eluent: hexane/EtOAc = 2:1) to afford a brown viscous oil (109 mg, 90%). ^1^H NMR (400 MHz, CDCl_3_) δ 7.49–7.39 (m, 4H, Ar), 6.75 (m, 1H, NH), 4.40 (t, *J* = 7.0 Hz, 2H, CH_2_), 4.02 (t, *J* = 6.5 Hz, 2H, CH_2_), 2.29 (q, *J* = 7.6 Hz, 2H, CH_2_), 2.03–1.92 (m, 2H, CH_2_), 1.64 (s, 6H, C(CH_3_)_2_), 1.62–1.53 (m, 2H, CH_2_), 1.52 (s, 9H, Boc), 1.45–1.32 (m, 4H, 2CH_2_), 1.11 (t, *J* = 7.6 Hz, 3H, CH_3_). ^13^C NMR (101 MHz, CDCl_3_) δ 174.7, 152.4, 140.2, 133.1, 132.8, 124.1, 118.3, 115.1, 103.1, 100.1, 81.4, 72.6, 71.6, 65.7, 64.2, 49.5, 31.4, 29.6, 28.6, 28.4, 27.7, 26.2, 25.5, 9.3. HSMS ESI: [M + Na]^+^ calcd. for C_29_H_38_N_4_O_5_Na^+^ 545.2734; found: 545.2732.

6-(4-(3-Hydroxy-3-methylbut-1-yn-1-yl)-5-((3,4,5-trimethoxyphenyl)ethynyl)-1*H*-1,2,3-triazol-1-yl)hexyl propionate (**7c**) was prepared in accordance with the general procedure from 5-iodo-1*H*-1,2,3-triazole **5b** (150 mg, 0.35 mmol), 5-ethynyl-1,2,3-trimethoxybenzene **6c** (73.2 mg, 0.38 mmol), Pd(PPh_3_)_4_ (20 mg, 0.017 mmol), CuI (6.6 mg, 0.035 mmol) and K_3_PO_4_ (80.8 mg, 0.38 mmol) with a reaction time of 6 h. The crude product was purified by column chromatography (eluent: hexane/EtOAc = 1:1) to afford a white solid (114 mg, 66%). ^1^H NMR (400 MHz, CDCl_3_) δ 6.76 (s, 2H, Ar), 4.41 (t, *J* = 7.0 Hz, 2H), 4.02 (t, *J* = 6.6 Hz, 2H), 3.90–3.85 (m, 9H, 3OMe), 2.28 (q, *J* = 7.6 Hz, 2H), 2.01–1.97 (m, *J* = 7.2 Hz, 2H), 1.64 (s, 6H), 1.62–1.53 (m, 2H), 1.42–1.34 (m, 4H), 1.10 (t, *J* = 7.5 Hz, 3H). ^13^C NMR (101 MHz, CDCl_3_) δ 174.7, 153.4, 140.4, 133.4, 123.9, 116.0, 109.2, 103.1, 100.2, 72.3, 71.5, 65.7, 64.1, 61.2, 56.4, 49.5, 31.3, 29.6, 28.5, 27.7, 26.2, 25.5, 9.2. HSMS ESI: [M + H]^+^ calc for C_27_H_35_N_3_O_6_Na^+^ 520.2418; found: 520.2415.

6-(4-(3-Hydroxy-3-methylbut-1-yn-1-yl)-5-((2,4,6-trimethoxyphenyl)ethynyl)-1*H*-1,2,3-triazol-1-yl)hexyl propionate (**7d**) was prepared in accordance with the general procedure from 5-iodo-1*H*-1,2,3-triazole **5b** (150 mg, 0.35 mmol), 2-ethynyl-1,3,5-trimethoxybenzene **6d** (73.2 mg, 0.38 mmol), Pd(PPh_3_)_4_ (20 mg, 0.017 mmol), CuI (6.6 mg, 0.035 mmol) and K_3_PO_4_ (80.8 mg, 0.38 mmol) with a reaction time of 6 h. The crude product was purified by column chromatography (eluent: hexane/EtOAc = 2:1) to afford a white solid (128 mg, 74%). ^1^H NMR (400 MHz, CDCl_3_) δ 6.11 (s, 2H, Ar), 4.43 (t, *J* = 7.0 Hz, 2H), 4.01 (t, *J* = 6.6 Hz, 2H), 3.87 (s, 6H, 2OMe), 3.84 (s, 3H, OMe), 2.38 (s, 1H, OH), 2.28 (q, *J* = 7.6 Hz, 2H), 2.08–1.91 (m, 2H), 1.63 (s, 6H), 1.63–1.52 (m, 2H), 1.43–1.29 (m, 4H), 1.10 (t, *J* = 7.6 Hz, 3H). ^13^C NMR (101 MHz, CDCl_3_) δ 174.7, 163.2, 162.7, 131.8, 125.3, 99.6, 96.9, 93.0, 90.7, 80.1, 72.1, 65.7, 64.3, 56.2, 55.7, 49.3, 31.3, 29.5, 28.6, 27.7, 26.2, 25.5, 9.2. HSMS ESI: [M + Na]^+^ calcd. for C_27_H_35_N_3_O_6_Na^+^ 520.2418; found: 520.2413.

4-(5-((4-(Dimethylamino)phenyl)ethynyl)-1-(4-methoxybenzyl)-1*H*-1,2,3-triazol-4-yl)-2-methylbut-3-yn-2-ol (**7e**) was prepared in accordance with the general procedure from 5-iodo-1*H*-1,2,3-triazole **5c** (100 mg, 0.25 mmol), 4-ethynyl-*N,N*-dimethylaniline **6a** (43.9 mg, 0.30 mmol), Pd(PPh_3_)_4_ (14.6 mg, 0.013 mmol), CuI (4.8 mg, 0.025 mmol) and K_3_PO_4_ (58.8 mg, 0.28 mmol) with a reaction time of 5 h. The crude product was purified by column chromatography (eluent: hexane/acetone = 2:1) to afford a brown solid (80 mg, 77%). ^1^H NMR (400 MHz, CDCl_3_) δ 7.44–7.37 (m, 2H, Ar), 7.37–7.30 (m, 2H, Ar), 6.95–6.83 (m, 2H, Ar), 6.73–6.65 (m, 2H, Ar), 5.52 (s, 2H, CH_2_), 3.80 (s, 3H, OMe), 3.05 (s, 6H, NMe_2_), 2.31 (s, 1H, OH), 1.65 (s, 6H, C(CH_3_)_2_). ^13^C NMR (101 MHz, CDCl_3_) δ 159.9, 151.1, 133.1, 132.7, 129.9, 126.8, 124.7, 114.7, 111.8, 107.5, 105.2, 99.8, 71.9, 71.8, 65.7, 55.4, 52.8, 40.2, 31.4. HSMS ESI: [M + H]^+^ calcd. for C_25_H_27_N_4_O_2_^+^ 415.2129; found: 415.2122.

*tert*-Butyl (4-((4-(3-hydroxy-3-methylbut-1-yn-1-yl)-1-(4-methoxybenzyl)-1*H*-1,2,3-triazol-5-yl)ethynyl)phenyl)carbamate (**7f**) was prepared in accordance with the general procedure from 5-iodo-1*H*-1,2,3-triazole **5c** (100 mg, 0.25 mmol), *tert*-butyl (4-ethynylphenyl)carbamate **6b** (65.4 mg, 0.30 mmol), Pd(PPh_3_)_4_ (14.6 mg, 0.013 mmol), CuI (4.8 mg, 0.025 mmol) and K_3_PO_4_ (58.8 mg, 0.28 mmol) with a reaction time of 5 h. The crude product was purified by column chromatography (eluent: hexane/acetone = 2:1) to afford a yellow solid. (103 mg, 84%). ^1^H NMR (400 MHz, CDCl_3_) δ 7.42 (s, 4H, Ar), 7.35–7.27 (m, 2H, Ar), 6.91–6.84 (m, 2H, Ar), 6.75 (br.s, 1H, NH), 5.51 (s, 2H, CH_2_), 3.77 (s, 3H, OMe), 1.62 (s, 6H, C(CH_3_)_2_), 1.52 (s, 9H, Boc). ^13^C NMR (101 MHz, CDCl_3_) δ 160.0, 152.4, 140.2, 132.8, 129.8, 126.5, 124.0, 118.3, 115.2, 114.4, 103.4, 100.1, 81.4, 72.9, 71.6, 65.7, 55.5, 53.0, 31.3, 28.4. HSMS ESI: [M + H]^+^ calcd. for C_28_H_31_N_4_O_4_^+^ 487.2340; found: 487.2336.

#### 3.2.3. General Procedure for the Synthesis of Terminal 4-Ethynyl-1,2,3-Triazoles **8a**–**f** by the Retro-Favorskii Reaction

A round-bottom oven-dried flask equipped with a magnetic stirring bar was charged with a solution of corresponding alcohol **7a**–**f** (1.00 equiv.) in dry benzene (2.0 mL) through the septum via syringe. Argon was bubbled through the solution for 10 min, and then well-ground anhydrous KOH (2.0 equiv. for **7c**,**d**,**f**, 3.0 equiv. for **7a**, and 4.0 equiv. for compound **7b**,**e**) was added in the stream of Ar. A reflux condenser was equipped, and the flask with the resulting mixture was heated on an oil bath (bath temperature 75 °C). After completion of the reaction (TLC control), the reaction mixture was cooled, and a precipitate was filtered through a short pad of silica gel eluting with benzene. The solvent was removed under reduced pressure, and the crude product was purified by column chromatography on silica gel using EtOAc/hexane as the eluent.

6-(5-((4-(Dimethylamino)phenyl)ethynyl)-4-ethynyl-1*H*-1,2,3-triazol-1-yl)hexyl propionate (**8a**) was prepared in accordance with the general procedure from **7a** (70 mg, 0.12 mmol) and KOH (20 mg, 0.36 mmol). The crude product was purified by column chromatography (eluent: hexane/acetone = 3:1), which afforded a brown viscous oil (35 mg, 57% yield). ^1^H NMR (400 MHz, CDCl_3_) δ 7.48–7.40 (m, 2H, Ar), 6.73–6.64 (m, 2H, Ar), 4.41 (t, *J* = 7.1 Hz, 2H, CH_2_), 4.04 (t, *J* = 6.6 Hz, 2H, CH_2_), 3.41 (s, 1H, CH), 3.02 (s, 6H, NMe_2_), 2.30 (q, *J* = 7.5 Hz, 2H, CH_2_), 2.05–1.91 (m, 2H, CH_2_), 1.67–1.58 (m, 2H, CH_2_), 1.46–1.36 (m, 4H, 2CH_2_), 1.12 (t, *J* = 7.5 Hz, 3H, CH_3_). ^13^C NMR (101 MHz, CDCl_3_) δ 174.7, 151.2, 133.2, 131.7, 125.6, 111.8, 107.2, 105.0, 83.2, 73.3, 71.3, 64.3, 49.3, 40.2, 29.6, 28.6, 27.7, 26.2, 25.6, 9.3. HSMS ESI: [M + H]^+^ calc C23H29N4O2^+^ 393.2285; found: 393.2286.

6-(5-((4-((*tert*-Butoxycarbonyl)amino)phenyl)ethynyl)-4-ethynyl-1*H*-1,2,3-triazol-1-yl)hexyl propionate (**8b**) was prepared in accordance with the general procedure from **7b** (100 mg, 0.19 mmol) and KOH (43 mg, 0.77 mmol). The crude product was purified by column chromatography (eluent: hexane/EtOAc = 3:1), which afforded a white solid (58 mg, 65%). ^1^H NMR (400 MHz, CDCl_3_) δ 7.52–7.39 (m, 4H, Ar), 6.70 (s, 1H, NH), 4.42 (t, *J* = 7.0 Hz, 2H, CH_2_), 4.03 (t, *J* = 6.6 Hz, 2H, CH_2_), 3.42 (s, 1H, CH), 2.30 (q, *J* = 7.6 Hz, 2H, CH_2_), 2.06–1.93 (m, 2H, CH_2_), 1.67–1.56 (m, 2H, CH_2_), 1.52 (s, 9H, Boc), 1.47–1.32 (m, 4H, CH_2_), 1.12 (t, *J* = 7.6 Hz, 3H, CH_3_). ^13^C NMR (101 MHz, CDCl_3_) δ 174.7, 152.4, 140.3, 132.9, 132.4, 124.9, 118.2, 115.0, 103.2, 83.5, 81.4, 73.0, 72.3, 64.2, 49.5, 29.7, 28.6, 28.3, 27.7, 26.2, 25.6, 9.3. HSMS ESI: [M + Na]^+^ calcd. for C_26_H_32_N_4_O_4_Na^+^ 487.2316; found: 487.2307.

6-(4-Ethynyl-5-((3,4,5-trimethoxyphenyl)ethynyl)-1*H*-1,2,3-triazol-1-yl)hexyl propionate (**8c**) was prepared in accordance with the general procedure from **7c** (50 mg, 0.10 mmol) and KOH (11.3 mg, 0.20 mmol). The crude product was purified by column chromatography (eluent: hexane/EtOAc = 3:1) to afford a white solid (32 mg, 73%). ^1^H NMR (400 MHz, CDCl_3_) δ 6.77 (s, 2H, Ar), 4.42 (t, *J* = 7.0 Hz, 2H, CH_2_), 4.03 (t, *J* = 6.6 Hz, 2H, CH_2_), 3.90–3.87 (m, 9H, 3OMe), 3.43 (s, 1H, CH), 2.33–2.25 (m, 2H, CH_2_), 2.03–1.95 (m, 2H, CH_2_), 1.67–1.56 (m, 2H, CH_2_), 1.43–1.35 (m, 4H, 2CH_2_), 1.13–1.07 (m, 3H, CH_3_). ^13^C NMR (101 MHz, CDCl_3_) δ 174.6, 153.4, 140.5, 132.6, 124.6, 115.8, 109.4, 103.1, 83.7, 72.9, 72.0, 64.1, 61.2, 56.4, 49.5, 29.6, 28.6, 27.7, 26.2, 25.5, 9.2. HSMS ESI: [M + Na]^+^ calcd. for C_24_H_29_N_3_O_5_Na^+^ 462.1999; found: 462.1996.

6-(4-Ethynyl-5-((2,4,6-trimethoxyphenyl)ethynyl)-1*H*-1,2,3-triazol-1-yl)hexyl propionate (**8d**) was prepared in accordance with the general procedure from **7d** (50 mg, 0.10 mmol) and KOH (11.3 mg, 0.20 mmol). The crude product was purified by column chromatography (eluent: hexane/EtOAc = 3:1) to afford a white solid (30 mg, 68%). ^1^H NMR (400 MHz, CDCl_3_) δ 6.11 (s, 2H, Ar), 4.44 (t, *J* = 7.1 Hz, 2H, CH_2_), 4.01 (t, *J* = 6.6 Hz, 2H, CH_2_), 3.87 (s, 6H, 2OMe), 3.84 (s, 3H, OMe), 3.40 (s, 1H, CH), 2.28 (q, *J* = 7.6 Hz, 2H, CH_2_), 2.05–1.95 (m, 2H, CH_2_), 1.65–1.54 (m, 2H, CH_2_), 1.42–1.34 (m, 4H, 2CH_2_), 1.10 (t, *J* = 7.6 Hz, 3H, CH_3_). ^13^C NMR (101 MHz, CDCl_3_) δ 174.6, 163.3, 162.8, 131.3, 125.8, 97.1, 92.8, 90.7, 83.0, 79.7, 73.3, 64.2, 56.2, 55.7, 49.3, 29.5, 28.6, 27.7, 26.2, 25.5, 9.2. HSMS ESI: [M + Na]^+^ calcd. for C_24_H_29_N_3_O_5_Na^+^ 462.1999; found: 462.1993.

4-((4-Ethynyl-1-(4-methoxybenzyl)-1*H*-1,2,3-triazol-5-yl)ethynyl)-*N,N*-dimethylaniline (**8e**) was prepared in accordance with the general procedure from **7e** (50 mg, 0.12 mmol) and KOH (27 mg, 0.48 mmol). The crude product was purified by column chromatography (eluent: hexane/acetone = 3:1) to afford a slightly yellow solid (28 mg, 65%). ^1^H NMR (400 MHz, CDCl_3_) δ 7.39 (d, *J* = 8.5 Hz, 2H, Ar), 7.33 (d, *J* = 8.3 Hz, 2H, Ar), 6.86 (d, *J* = 8.3 Hz, 2H, Ar), 6.66 (d, *J* = 8.5 Hz, 2H, Ar), 5.50 (s, 2H, CH_2_), 3.78 (s, 3H, OMe), 3.39 (s, 1H, CH), 3.02 (s, 6H, NMe_2_). ^13^C NMR (101 MHz, CDCl_3_) δ 159.9, 151.2, 133.2, 132.0, 129.9, 126.7, 125.4, 114.3, 111.7, 107.2, 105.3, 83.3, 73.2, 71.5, 55.4, 52.7, 40.2. HSMS ESI: [M + H]^+^ calcd. for C_22_H_21_N_4_O^+^ 357.1710; found: 357.1712.

*tert*-Butyl (4-((4-ethynyl-1-(4-methoxybenzyl)-1*H*-1,2,3-triazol-5-yl)ethynyl)phenyl)carbamate (**8f**) was prepared in accordance with the general procedure from **7f** (50 mg, 0.10 mmol) and KOH (11.5 mg, 0.21 mmol). The crude product was purified by column chromatography (eluent: hexane/EtOAc = 3:1) to afford a white solid (40 mg, 91%). ^1^H NMR (400 MHz, CDCl_3_) δ 7.53–7.40 (m, 4H, Ar), 7.34–7.27 (m, 2H, Ar), 6.88–6.82 (m, 2H, Ar), 6.75 (s, 1H, NH), 5.52 (s, 2H, CH_2_), 3.77 (s, 3H, OMe), 3.40 (s, 1H, CH), 1.52 (s, 9H, Boc). ^13^C NMR (101 MHz, CDCl_3_) δ 160.0, 152.4, 140.3, 132.9, 132.7, 129.9, 126.4, 124.7, 118.2, 115.0, 114.4, 103.5, 83.6, 81.4, 72.9, 72.5, 55.4, 53.0, 28.4. HSMS ESI: [M + Na]^+^ calcd. for C_25_H_24_N_4_O_3_Na^+^ 451.1741; found: 451.1738.

#### 3.2.4. General Procedure for the Synthesis of Trz-OAEs **10** by Sonogashira Cross-Coupling

Dietynyltriazole **8a**–**f** (1 equiv.), 1,4-diiodo-2,5-bis(alkyloxy)benzene **9a**,**b** (0.5 equiv.), CuI (3 mol%) and Pd(PPh_3_)_4_ (3 mol%) were placed in a vial. The vial was sealed, and the mixture was evacuated and flushed with argon several times. Diisopropilamine (DIPA) (40 equiv.) and dry toluene (0.03M) were added. The vial with the reaction mixture was placed in a pre-heated aluminum block at 40 °C and stirred at this temperature for 2–4 h (TLC control). After completion of the reaction, the reaction mixture was cooled, poured into a saturated aqueous solution of NH_4_Cl, and extracted with DCM. The combined organic layers were washed with a saturated solution of NH_4_Cl, two times with EDTA, and two times with brine, dried over anhydrous Na_2_SO_4_, and concentrated under reduced pressure to give the crude product, which was purified by column chromatography on silica gel.

(((2,5-Bis(octyloxy)-1,4-phenylene)bis(ethyne-2,1-diyl))bis(5-((4-(dimethylamino)phenyl)ethynyl)-1*H*-1,2,3-triazole-4,1-diyl))bis(hexane-6,1-diyl) dipropionate (**10a**) was synthesized in accordance with the general procedure from alkyne **8a** (35 mg, 0.0892 mmol), 1,4-diiodobenzene **9a** (26.1 mg, 0.0446 mmol), Pd(PPh_3_)_4_ (3.1 mg, 0.0027 mmol), CuI (0.5 mg, 0.0027 mmol) and DIPA (0.500 mL, 3.57 mmol) with a reaction time of 2 h. The crude product was purified by recrystallization from acetonitrile/water (50:1) to afford **10a** as a yellow solid (30 mg, 60%). ^1^H NMR (400 MHz, CDCl_3_) δ 7.46–7.37 (m, 4H, Ar), 7.08 (s, 2H, Ar), 6.69–6.61 (m, 4H, Ar), 4.43 (t, *J* = 7.0 Hz, 4H, 2CH_2_), 4.04 (t, *J* = 6.5 Hz, 4H, 2CH_2_), 3.98 (t, *J* = 6.8 Hz, 4H, 2CH_2_), 3.02 (s, 12H, 2NMe_2_), 2.30 (q, *J* = 7.6 Hz, 4H, 2CH_2_), 2.06–1.94 (m, 4H, 2CH_2_), 1.74 (p, *J* = 7.0 Hz, 4H, 2CH_2_), 1.66–1.58 (m, 4H, 2CH_2_), 1.48–1.31 (m, 12H, 6CH_2_), 1.28–1.17 (m, 14H, 7CH_2_), 1.12 (t, *J* = 7.6 Hz, 6H, 2CH_3_), 0.83 (t, *J* = 6.8 Hz, 6H, 2CH_3_). ^13^C NMR (101 MHz, CDCl_3_) δ 174.7, 153.8, 151.1, 133.2, 133.0, 124.7, 117.7, 114.0, 111.7, 107.5, 105.0, 91.6, 84.2, 71.8, 70.1, 64.3, 49.3, 40.2, 31.9, 29.7, 29.38, 29.37, 29.3, 28.6, 27.7, 26.2, 26.0, 25.6, 22.8, 14.2, 9.3. HSMS ESI: [M + H]^+^ calcd. for C_68_H_91_N_8_O_6_^+^ 1115.7056; found: 1115.7057.

(((2,5-Bis(octyloxy)-1,4-phenylene)bis(ethyne-2,1-diyl))bis(5-((4-((*tert*-butoxycarbonyl)amino)phenyl)ethynyl)-1*H*-1,2,3-triazole-4,1-diyl))bis(hexane-6,1-diyl) dipropionate (**10b**) was synthesized in accordance with the general procedure from alkyne **8b** (58 mg, 0.125 mmol), 1,4-diiodobenzene **9a** (36.6 mg, 0.0624 mmol), Pd(PPh_3_)_4_ (4.3 mg, 0.0038 mmol), CuI (0.7 mg, 0.0038 mmol) and DIPA (0.700 mL, 5 mmol) with a reaction time of 2 h. The crude product was purified by column chromatography (eluent: hexane/EtOAc = 2:1→1:1) to afford a yellow solid (67 mg, 85%). ^1^H NMR (400 MHz, CDCl_3_) δ 7.52–7.46 (m, 4H, Ar), 7.47–7.39 (m, 4H, Ar), 7.07 (s, 2H, Ar), 6.66 (s, 2H, 2 NH), 4.44 (t, *J* = 7.0 Hz, 4H, 2CH_2_), 4.04 (t, *J* = 6.6 Hz, 4H, 2CH_2_), 3.966 (t, *J* = 6.8 Hz, 4H, 2CH_2_), 2.30 (q, *J* = 7.6 Hz, 4H, 2CH_2_), 2.05–1.95 (m, 4H, 2CH_2_), 1.71 (p, *J* = 6.8 Hz, 4H, 2CH_2_), 1.66–1.56 (m, 4H, 2CH_2_), 1.55 (s, 18H, 2Boc), 1.45–1.31 (m, 14H, 2CH_2_), 1.29–1.15 (m, 14H, 2CH_2_), 1.12 (t, *J* = 7.6 Hz, 6H, 2CH_3_), 0.83 (t, *J* = 6.8 Hz, 6H, 2CH_3_). ^13^C NMR (101 MHz, CDCl_3_) δ 174.7, 153.8, 152.3, 140.2, 133.7, 132.9, 123.9, 118.1, 117.7, 115.3, 114.0, 103.1, 91.9, 83.9, 81.4, 72.9, 70.0, 64.2, 49.5, 31.9, 29.7, 29.4, 29.3, 28.6, 28.4, 27.7, 26.3, 26.0, 25.6, 22.8, 14.2, 9.3. HSMS ESI: [M + Na]^+^ calcd. for C_74_H_98_N_8_O_10_Na^+^ 1281.7298; found: 1281.7304.

(((2,5-Bis(octyloxy)-1,4-phenylene)bis(ethyne-2,1-diyl))bis(5-((3,4,5-trimethoxyphenyl)ethynyl)-1*H*-1,2,3-triazole-4,1-diyl))bis(hexane-6,1-diyl) dipropionate (**10c**) was synthesized in accordance with the general procedure from alkyne **8c** (45 mg, 0.103 mmol), 1,4-diiodobenzene **9a** (30 mg, 0.0512 mmol), Pd(PPh_3_)_4_ (3.6 mg, 0.0031 mmol), CuI (0.6 mg, 0.0031 mmol) and DIPA (0.574 mL, 4.1 mmol) with a reaction time of 2 h. The crude product was purified by column chromatography (eluent: hexane/EtOAc = 2:1→1:1) to afford a white solid (41 mg, 66%). ^1^H NMR (400 MHz, CDCl_3_) δ 7.07 (s, 2H, Ar), 6.79 (s, 4H, Ar), 4.45 (t, *J* = 7.0 Hz, 4H, 2CH_2_), 4.04 (t, *J* = 6.6 Hz, 4H, 2CH_2_), 3.96 (t, *J* = 6.7 Hz, 4H, 2CH_2_), 3.89 (s, 6H, 2OMe), 3.87 (s, 12H, 4OMe), 2.29 (q, *J* = 7.6 Hz, 4H, 2CH_2_), 2.07–1.96 (m, 4H, 2CH_2_), 1.75–1.57 (m, 8H, 4CH_2_), 1.47–1.28 (m, 12H, 6CH_2_), 1.24–1.15 (m, 14H, 7CH_2_), 1.11 (t, *J* = 7.6 Hz, 6H, 2CH_3_), 0.83 (t, *J* = 6.9 Hz, 6H, 2CH_3_). ^13^C NMR (101 MHz, CDCl_3_) δ 174.7, 154.0, 153.4, 140.5, 133.9, 123.7, 118.0, 116.1, 114.2, 109.4, 103.2, 91.8, 83.9, 72.6, 70.3, 64.2, 61.2, 56.4, 49.5, 31.9, 29.7, 29.3, 29.3, 29.3, 28.6, 27.7, 26.3, 26.0, 25.6, 22.7, 14.2, 9.3. HSMS ESI: [M + H]^+^ calcd. for C_70_H_92_N_6_O_12_Na^+^ 1231.6665; found: 1231.6664.

(((2,5-Bis(octyloxy)-1,4-phenylene)bis(ethyne-2,1-diyl))bis(5-((2,4,6-trimethoxyphenyl)ethynyl)-1*H*-1,2,3-triazole-4,1-diyl))bis(hexane-6,1-diyl) dipropionate (**10d**) was synthesized in accordance with the general procedure from alkyne **8d** (67 mg, 0.152 mmol), 1,4-diiodobenzene **9a** (44.7 mg, 0.0762 mmol), Pd(PPh_3_)_4_ (5.3 mg, 0.0046 mmol), CuI (0.9 mg, 0.0046 mmol), DIPA (0.855 mL, 6.1 mmol) in dry toluene (3.0 mL) with a reaction time of 2 h. The crude product was purified by column chromatography (eluent: hexane/EtOAc = 1:1→1:2) to afford a white solid (41 mg, 45%). ^1^H NMR (400 MHz, CDCl_3_) δ 7.08 (s, 2H, Ar), 6.11 (s, 4H, Ar), 4.46 (t, *J* = 7.0 Hz, 4H, 2CH_2_), 4.03 (t, *J* = 6.6 Hz, 4H, 2CH_2_), 3.97 (t, *J* = 6.7 Hz, 4H, 2CH_2_), 3.87–3.80 (m, 6H, 2OMe), 3.83 (s, 12H, 4OMe), 2.29 (q, *J* = 7.6 Hz, 4H, 2CH_2_), 2.08–1.95 (m, 4H, 2CH_2_), 1.72 (p, *J* = 6.9 Hz, 4H, 2CH_2_), 1.66–1.56 (m, 4H, 2CH_2_), 1.44–1.30 (m, 12H, 6CH_2_), 1.27–1.16 (m, 14H, 7CH_2_), 1.12 (t, *J* = 7.6 Hz, 6H, 2CH_3_), 0.86 (t, *J* = 6.9 Hz, 6H, 2CH_3_). ^13^C NMR (101 MHz, CDCl_3_) δ 174.7, 163.2, 162.8, 154.0, 132.6, 125.1, 118.5, 114.5, 97.0, 93.2, 91.4, 90.7, 84.3, 80.3, 70.5, 64.3, 56.2, 55.7, 49.3, 32.0, 29.5, 29.4, 29.34, 29.30, 28.6, 27.7, 26.2, 26.0, 25.6, 22.8, 14.2, 9.3. HSMS ESI: [M + Na]^+^ calcd. for C_70_H_92_N_6_O_12_Na^+^ 1231.6665; found: 1231.6671.

4,4′-((((2,5-Bis(octyloxy)-1,4-phenylene)bis(ethyne-2,1-diyl))bis(1-(4-methoxybenzyl)-1*H*-1,2,3-triazole-4,5-diyl))bis(ethyne-2,1-diyl))bis(*N,N*-dimethylaniline) (**10e**) was synthesized in accordance with the general procedure from alkyne **8e** (35 mg, 0.098 mmol), 1,4-diiodobenzene **9a** (28.8 mg, 0.0491 mmol), Pd(PPh_3_)_4_ (3.4 mg, 0.003 mmol), CuI (0.6 mg, 0.003 mmol), DIPA (0.550 mL, 3.93 mmol) in dry toluene 1.6 mL with a reaction time of 2 h. The crude product was purified by recrystallization from acetonitrile, to give a yellow solid (35 mg, 68%). ^1^H NMR (400 MHz, CDCl_3_) δ 7.43–7.37 (m, 4H, Ar), 7.37–7.30 (m, 4H, Ar), 7.05 (s, 2H, Ar), 6.91–6.82 (m, 4H, Ar), 6.69–6.61 (m, 4H, Ar), 5.53 (s, 4H, 2CH_2_), 3.96 (t, *J* = 6.8 Hz, 4H, 2CH_2_), 3.78 (s, 6H, 2OMe), 3.02 (s, 12H, 2NMe_2_), 1.72 (p, *J* = 7.0 Hz, 4H, 2CH_2_), 1.40–1.29 (m, 4H, 2CH_2_), 1.27–1.12 (m, 16H, 8CH_2_), 0.83 (t, *J* = 6.8 Hz, 6H, 2CH_3_). ^13^C NMR (101 MHz, CDCl_3_) δ 159.9, 153.8, 151.1, 133.3, 133.2, 129.9, 126.9, 124.5, 117.8, 114.3, 114.1, 111.7, 107.6, 105.3, 91.7, 84.1, 72.1, 70.1, 55.4, 52.7, 40.2, 31.9, 29.4, 29.3, 26.0, 22.8, 14.2. HSMS ESI: [M + H]^+^ calcd. for C_66_H_75_N_8_O_4_^+^ 1043.5906; found: 1043.5911.

Di-*tert*-butyl (((((2,5-bis(octyloxy)-1,4-phenylene)bis(ethyne-2,1-diyl))bis(1-(4-methoxybenzyl)-1*H*-1,2,3-triazole-4,5-diyl))bis(ethyne-2,1-diyl))bis(4,1-phenylene))dicarbamate (**10′f**) was synthesized in accordance with the general procedure from alkyne **8f** (33 mg, 0.077 mmol), 1,4-diiodobenzene **9a** (22.6 mg, 0.0385 mmol), Pd(PPh_3_)_4_ (2.7 mg, 0.0023 mmol), CuI (0.4 mg, 0.0023 mmol), DIPA (0.432 mL, 3.08 mmol) in dry toluene 1.5 mL with a reaction time of 2 h. After removal of the solvent under reduced pressure, the product obtained was a yellow powder (41 mg, 90%), which we used without additional purification in the next step.

4,4′-((((2,5-Bis(octyloxy)-1,4-phenylene)bis(ethyne-2,1-diyl))bis(1-(4-methoxybenzyl)-1*H*-1,2,3-triazole-4,5-diyl))bis(ethyne-2,1-diyl))dianiline (**10f**). Compound **10′f** (40 mg, 0.0337 mmol) was dissolved in dry DCM (1 mL), and TFA (0.150 mL, 2.0 mmol) was added. The reaction mixture was stirred at room temperature for 3 h (TLC control) and then quenched by Na_2_CO_3_ (3 mL). The resulting mixture was extracted with DCM (3 × 5 mL). The combined organic layer was washed with brine (5 mL), dried over Na_2_SO_4_, filtered, and concentrated in vacuo. The crude product was purified by column chromatography (eluent: hexane/EtOAc = 1:1→1:2) to afford a white solid (14 mg, 42%). ^1^H NMR (400 MHz, DMSO-d_6_) δ 7.35–7.28 (m, 4H, Ar), 7.28–7.22 (m, 4H, Ar), 7.19 (s, 2H, Ar), 6.98–6.92 (m, 4H, Ar), 6.63–6.55 (m, 4H, Ar), 5.83 (s, 4H), 5.61 (s, 4H, 2CH_2_), 4.02 (t, *J* = 6.4 Hz, 4H, 2CH_2_), 3.73 (s, 6H, 2OMe), 1.63 (p, *J* = 6.8 Hz, 4H, 2CH_2_), 1.40–1.29 (m, 4H, 2CH_2_), 1.24–1.07 (m, 16H, 8CH_2_), 0.75 (t, *J* = 6.6 Hz, 6H, 2CH_3_). ^13^C NMR (101 MHz, DMSO-d_6_) δ 159.3, 153.1, 151.0, 133.1, 131.4, 129.5, 127.0, 124.2, 117.0, 114.2, 113.5, 113.0, 105.9, 105.5, 91.1, 84.0, 70.9, 69.1, 55.1, 52.0, 31.1, 28.6, 28.5, 25.3, 22.0, 13.9. HSMS ESI: [M + H]^+^ calcd. for C_62_H_67_N_8_O_4_^+^ 987.5280; found: 987.5296.

(((2,5-Dimethoxy-1,4-phenylene)bis(ethyne-2,1-diyl))bis(5-((4-(dimethylamino)phenyl)ethynyl)-1*H*-1,2,3-triazole-4,1-diyl))bis(hexane-6,1-diyl) dipropionate (**10g**) was synthesized in accordance with the general procedure from alkyne **8g** (58 mg, 0.148 mmol), 1,4-diiodo-2,5-dimethoxybenzene **9b** (28.8 mg, 0.0739 mmol), Pd(PPh_3_)_4_ (5.1 mg, 0.0044 mmol), CuI (0.8 mg, 0.0044 mmol), DIPA (0.828 mL, 5.9 mmol) in dry toluene 2.9 mL with a reaction time of 2 h. The crude product was purified by column chromatography (eluent: hexane/EtOAc = 2:1) to afford a white solid (47 mg, 69%). ^1^H NMR (400 MHz, CDCl_3_) δ 7.47–7.39 (m, 4H, Ar), 7.08 (s, 2H, Ar), 6.70–6.62 (m, 4H, Ar), 4.43 (t, *J* = 7.0 Hz, 4H, 2CH_2_), 4.04 (t, *J* = 6.5 Hz, 4H, 2CH_2_), 3.84 (s, 6H, 2OMe), 3.02 (s, 12H, 2NMe_2_), 2.30 (q, *J* = 7.6 Hz, 4H, 2CH_2_), 2.06–1.94 (m, 4H, 2CH_2_), 1.68–1.56 (m, 4H, 2CH_2_), 1.44–1.36 (m, 8H, 4CH_2_), 1.12 (t, *J* = 7.5 Hz, 6H, 2CH_3_). ^13^C NMR (101 MHz, CDCl_3_) δ 174.7, 154.2, 151.1, 133.2, 133.0, 125.0, 115.9, 113.4, 111.7, 107.5, 105.2, 91.5, 84.5, 71.8, 64.3, 56.7, 49.3, 40.2, 29.7, 28.6, 27.7, 26.2, 25.6, 9.3. HSMS ESI: [M + Na]^+^ calcd. for C_54_H_62_N_8_O_6_Na^+^ 941.4685; found: 941.4698.

#### 3.2.5. General Procure for the Synthesis of Trz-OAEs **11**

To a stirred solution of triazoles **10** (1 equiv.) in 5 mL of THF/H_2_O/EtOH (3:1:1) an aqueous solution of LiOH × H_2_O (20 equiv., 2M) was added, and the reaction mixture was stirred at room temperature for 4 h; the reaction mixture was poured into a saturated aqueous solution of NH_4_Cl and extracted with DCM. The combined extracts were washed with brine (1 × 10 mL) and dried with Na_2_SO_4_, and concentrated under reduced pressure to give the crude product. The formed Trz-OAEs **11** were then dissolved in THF (1 mL) and precipitated in cold water. The precipitate was filtered, washed with water, and dried under vacuum.

6,6′-(((2,5-Bis(octyloxy)-1,4-phenylene)bis(ethyne-2,1-diyl))bis(5-((4-(dimethylamino)phenyl)ethynyl)-1*H*-1,2,3-triazole-4,1-diyl))bis(hexan-1-ol) (**11a**) was prepared in accordance with the general procedure from **10a** (75 mg, 68.2 mmol), LiOH × H_2_O (0.682 mL, 2M) in THF/H_2_O/EtOH (2.4 mL/0.8 mL/0.8 mL). The final product **11a** was obtained as a yellow solid (47 mg, 70%). ^1^H NMR (400 MHz, CDCl_3_) δ 7.46–7.36 (m, 4H, Ar), 7.08 (s, 2H, Ar), 6.69–6.64 (m, 4H, Ar), 4.43 (t, *J* = 7.0 Hz, 4H, 2CH_2_), 3.98 (t, *J* = 6.8 Hz, 4H, 2CH_2_), 3.61 (t, *J* = 6.5 Hz, 4H, 2CH_2_), 3.01 (s, 12H, 2NMe_2_), 2.00 (p, *J* = 7.0 Hz, 4H, 2CH_2_), 1.74 (p, *J* = 7.0 Hz, 4H, 2CH_2_), 1.62–1.51 (m, 4H, 2CH_2_), 1.49–1.31 (m, 12H, 6CH_2_), 1.23–1.14 (m, 14H, 7CH_2_), 0.83 (t, *J* = 6.8 Hz, 6H, 2CH_3_). ^13^C NMR (101 MHz, CDCl_3_) δ 153.7, 151.1, 133.2, 133.0, 124.7, 117.7, 114.0, 111.7, 107.5, 104.9, 91.6, 84.2, 71.8, 70.1, 62.8, 49.3, 40.2, 32.6, 31.9, 29.8, 29.7, 29.38, 29.36, 29.3, 26.3, 26.0, 25.2, 22.8, 14.2. HSMS ESI: [M + H]^+^ calcd. for C_62_H_83_N_8_O_4_^+^ 1003.6532; found: 1003.6526.

6,6′-(((2,5-Bis(octyloxy)-1,4-phenylene)bis(ethyne-2,1-diyl))bis(5-((3,4,5-trimethoxyphenyl)ethynyl)-1*H*-1,2,3-triazole-4,1-diyl))bis(hexan-1-ol) (**11c**) was prepared in accordance with general procedure from **10c** (35 mg, 28.9 mmol), LiOH × H_2_O (0.289 mL, 2M) in THF/H_2_O/EtOH (1.2 mL/0.4 mL/0.4 mL). The final product **11c** was obtained as a white solid (25 mg, 78%). ^1^H NMR (400 MHz, CDCl_3_) δ 7.07 (s, 2H, Ar), 6.79 (s, 4H, Ar), 4.45 (t, *J* = 7.0 Hz, 4H, 2CH_2_), 3.96 (t, *J* = 6.7 Hz, 4H, 2CH_2_), 3.88 (s, 6H, OMe), 3.86 (s, 12H, 2OMe), 3.62 (t, *J* = 6.5 Hz, 4H, 2CH_2_), 2.02 (p, *J* = 7.1 Hz, 4H, 2CH_2_), 1.75–1.63 (m, 4H, 2CH_2_), 1.56 (p, *J* = 6.7 Hz, 4H, 2CH_2_), 1.48–1.30 (m, 12H, 6CH_2_), 1.21–1.14 (m, 14H, 7CH_2_), 0.82 (t, *J* = 6.9 Hz, 6H, 2CH_3_). ^13^C NMR (101 MHz, CDCl_3_) δ 154.0, 153.4, 140.5, 133.8, 123.7, 117.9, 116.1, 114.2, 109.4, 103.1, 91.8, 83.9, 72.6, 70.3, 62.7, 61.2, 56.4, 49.6, 32.5, 31.9, 29.8, 29.7, 29.30, 29.28, 29.25, 26.3, 25.9, 25.3, 22.7, 14.2. HSMS ESI: [M + Na]^+^ calcd. for C_64_H_84_N_6_O_10_Na^+^ 1119.6141; found: 1119.6132.

6,6′-(((2,5-Bis(octyloxy)-1,4-phenylene)bis(ethyne-2,1-diyl))bis(5-((2,4,6-trimethoxyphenyl)ethynyl)-1*H*-1,2,3-triazole-4,1-diyl))bis(hexan-1-ol) (**11d**) was prepared in accordance with general procedure from **10d** (35 mg, 28.9 mmol), LiOH × H_2_O (0.289 mL, 2M) in THF/H_2_O/EtOH (1.2 mL/0.4 mL/0.4 mL). The final product **11d** was obtained as a white solid (28 mg, 91%). ^1^H NMR (400 MHz, CDCl_3_) δ 7.07 (s, 2H, Ar), 6.10 (s, 4H, Ar), 4.47 (t, *J* = 7.1 Hz, 4H, 2CH_2_), 3.97 (t, *J* = 6.7 Hz, 4H, 2CH_2_), 3.84 (s, 6H, 2OMe), 3.85 (s, 12H, 4OMe), 3.60 (t, *J* = 6.4 Hz, 4H, 2CH_2_), 2.02 (p, *J* = 7.0 Hz, 4H, 2CH_2_), 1.78–1.66 (m, 4H, 2CH_2_), 1.61–1.50 (m, 4H, 2CH_2_), 1.47–1.33 (m, 12H, 6CH_2_), 1.20–1.15 (m, 14H, 7CH_2_), 0.83 (t, *J* = 6.9 Hz, 6H, 2CH_3_). ^13^C NMR (101 MHz, CDCl_3_) δ 163.2, 162.8, 154.0, 132.6, 125.1, 118.5, 114.5, 97.1, 93.2, 91.4, 90.7, 84.3, 80.3, 70.5, 62.8, 56.2, 55.6, 49.3, 32.6, 31.9, 29.6, 29.4, 29.33, 29.29, 26.2, 26.0, 25.2, 22.8, 14.2. HSMS ESI: [M + Na]^+^ calcd. for C_64_H_84_N_6_O_10_Na^+^ 1119.6141; found: 1119.6131.

6,6′-(((2,5-Dimethoxy-1,4-phenylene)bis(ethyne-2,1-diyl))bis(5-((4-(dimethylamino)phenyl)ethynyl)-1*H*-1,2,3-triazole-4,1-diyl))bis(hexan-1-ol) (**11g**) was prepared in accordance with general procedure from **10g** (40 mg, 43.5 mmol), LiOH × H_2_O (0.435 mL, 2M) in THF/H_2_O/EtOH (1.5 mL/0.5 mL/0.5 mL). The final product, **11g**, was obtained as a yellow solid (33 mg, 94%). ^1^H NMR (400 MHz, CDCl_3_) δ 7.48–7.43 (m, 4H, Ar), 7.08 (s, 2H, Ar), 6.70–6.62 (m, 4H, Ar), 4.44 (t, *J* = 7.0 Hz, 4H, 2CH_2_), 3.84 (s, 6H, 2OMe), 3.62 (t, *J* = 6.4 Hz, 4H, 2CH_2_), 3.02 (s, 12H, 2NMe_2_), 2.01 (p, *J* = 7.1 Hz, 4H, 2CH_2_), 1.57 (p, *J* = 6.6 Hz, 4H, 2CH_2_), 1.48–1.34 (m, 8H, 2CH_2_). ^13^C NMR (101 MHz, CDCl_3_) δ 154.3, 151.1, 133.2, 133.0, 125.0, 116.0, 113.4, 111.8, 107.5, 105.2, 91.5, 84.5, 71.9, 62.8, 56.7, 49.4, 40.2, 32.6, 29.7, 26.2, 25.2. HSMS ESI: [M + H]^+^ calcd. for C_48_H_55_N_8_O_4_^+^ 807.4341; found: 807.4352.

## 4. Conclusions

In this study, we demonstrated the effectiveness of the *retro*-Favorskii reaction for the synthesis of 4,5-(dialkynyl) triazoles with a terminal triple bond at the 4-th position. We employed this methodology to obtain a series of fluorescent oligo(arylene ethynylene)s of the D-π-A type, which include a two-electron-deficient 1,2,3-triazole core as a structural element in the main chain and aryl moieties with electron-donating substituents linked by a phenylene π-spacer.

Trz-OAEs with trimethoxyphenyl substituents exhibit the most intense fluorescence with the highest quantum yield up to 88%. For compounds with trimethoxyphenyl substituents, no noticeable change in quantum yields is observed, and the Stokes shift practically does not change with increasing polarity. At the same time, for OAEs bearing para-aminophenyl moieties at the 5-position of the triazole ring, an increase in Stokes shifts and a significant decrease in quantum yields were observed. This large Stokes’ shift indicates extensive intramolecular charge transfer across the molecule, which might be due to strong interactions between the polar solvent and the amino groups in the excited state. The decrease in quantum yields in the case of OAEs with amino groups is most likely due to curved intramolecular charge transfer in polar solvents and interaction with solvent through hydrogen bonds, which possibly enhance nonradiative processes, leading to fluorescence quenching.

The emission spectra obtained for derivatives containing para-dimethylaminoarylethynyl moieties in aqueous DMSO revealed an aggregate-induced emission enhancement. Dynamic light scattering (DLS) data also confirmed the formation of nanoscale aggregates in aqueous-organic solutions. The largest aggregates were found in DMSO with 30% water, which correlates with the maximum increase in emission intensity.

The tunable photoluminescence and AIEE properties of triazole-based OAEs make them promising candidates for the development of fluorescent dyes and probes for bioimaging. However, further research is needed to fully understand the relationship between their structural parameters and their fluorescence properties in different environments, and these studies are ongoing.

## Figures and Tables

**Table 1:** Photophysical parameters of Trz-OAEs **10a**,**c**–**g**.

Compound	λ_abs_, nm (ε (cm^−1^M^−1^))	λ_em_, nm	Stokes Shift, nm/cm^−1^	τ, ns	Φ_F_,% ^1^	Eg^opt^, eV ^2^
**10a**	308 (62,721) 347 (53,439)	424	77/5234	2.94	32	2.95
**10c**	289 (45,343) 375 (28,473)	412	37/2395	2.3	88	3.01
**10d**	293 (72,046) 335 (54,132) 363 (42,338)	411 433	48/3217	2.3	63	2.98
**10e**	308 (58,169) 346 (49,455)	427 449	80/5428	3.11	42	2.94
**10f**	302 (51,292) 337 (41,258) 375 (30,807)	418 438	43/2924	2.35	50	2.91
**10g**	307 (66,455) 346 (57,730)	455	109/6938	3.62	56	2.95

^1^ Absolute fluorescence quantum yield determined by a calibrated integrating sphere system; ^2^ Eg^opt^ was calculated on the direct application of the Tauc plot.

**Table 2:** Photophysical parameters **10a**–**g** in various solvents.

Compound	Solvent	λ_abs_, nm (ε (cm^−1^M^−1^))	λ_em_, nm	Stokes Shift, nm/cm^−1^	Φ_F_, %	τ, ns
**10a**	THF	308 (62,721); 347 (53,439)	424	77/5234	32	2.94
	*i*-PrOH	308 (52,396); 347 (43,492)	473	126/7677	35	3.92
	MeCN	308 (70,197); 350 (58,382)	527	177/9596	11	4.02
	DMSO	312 (64,502); 354 (55,128)	538	184/9661	7	2.67
	Water	312 (38,655); 352 (43,359)	473	121/7267	9	1.11 (74%) 5.49 (26%) [2.27]
**10c**	THF	289 (45,343); 375 (28,473)	412	37/2395	88	1.98
	*i*-PrOH	289 (53,680); 375 (28,180)	411	36/2348	66	2.06
	MeCN	288 (47,709); 374 (29,463)	413	39/2395	75	2.11
	DMSO	292 (44,554); 379 (27,329)	418	39/2461	83	1.11 (77%) 2.28 (23%) [1.38]
	Water	295 (39,152) 333 (40,596) 389 (34,755)	424	35/2112	15	0.80 (93%) 2.86 (7%) [0.95]
**10d**	THF	293 (72,046); 335 (54,132) 363 (42,338)	411	48/3217	63	1.73
	*i*-PrOH	293 (73,538); 333 (58,360); 362 (41,748)	411 433	49/4386	50	1.82
	MeCN	293 (62,209); 335 (54,195); 363 (40,596)	412 433	49/4454	78	1.82
	DMSO	296 (61,151); 339 (52,618); 369 (39,549)	419 440	50/3249	65	1.66
	Water	297 (58,537); 339 (51,019) 369 (37,951)	421 445	52/4628	13	0.72 (96%) 3.24 (4%) [0.82]
**10e**	THF	308 (58,169); 346 (49,455)	427 449	80/5428	42	3.11
	*i*-PrOH	307 (53,375); 346 (43,673)	479	133/8069	26	3.91
	MeCN	308 (47,271); 350 (39,717)	525	175/9560	11	4.03
	DMSO	313 (57,801); 354 (50,254)	547	193/10,003	7	2.64
	Water	314 (37,667); 354 (32,464)	470	116/7011	9	1.90 (51%) 7.59 (49%) [4.69]
**10f**	THF	302 (51,292); 337 (41,258); 375 (30,807)	418; 438	43/2924	50	2.35
	*i*-PrOH	303 (52,023); 337 (40,779)	481	144/8534	18	2.65
	MeCN	300 (54,611); 337 (41,377); 375 (30,307)	503	128/7270	12	1.99
	DMSO	308 (54,205); 350 (44,809)	527	177/11,700	0.2	9.82
	Water	305 (47,585); 342 (37,907)	468	126/7918	1	0.69 (76%) 3.31 (24%) [1.32]
**10g**	THF	307 (66,455); 346 (57,730)	455	109/6938	56	3.25
	*i*-PrOH	307 (66,305); 347 (56,260)	490	143/8497	30	4.14
	MeCN	307 (64,834); 350 (55,202)	537	187/9959	12	3.31
	DMSO	312 (61,668); 352 (54,256)	553	201/13,082	7	1.02
	Water	311 (53,624); 348 (43,247)	486	138/8192	16	3.27 (61%) 13.41 (29%) [7.18]

**Table 3:** Photophysical parameters **11a**,**c**,**d**,**g** in various solvents.

Compound	Solvent	λ_abs_, nm (ε (cm^−1^M^−1^))	λ_em_, nm	Stokes Shift, nm/cm^−1^	Φ_F_, %	Eg^opt^, eV ^1^
**11a**	THF	307 (65,565); 345 (56,724)	424	79/5400	48	2.98
	*i*-PrOH	307 (66,985); 347 (55,979)	472	125/7622	42	2.89
	MeCN	307 (63,764); 347 (54,277)	531	184/9986	13	2.97
	DMSO	312 (62,765); 354 (54,518)	541	187/12,399	14	2.94
	H_2_O	316 (51,752); 349 (45,650)	476	127/7613	4	2.97
**11c**	THF	289 (55,379); 375 (35,094)	412	37/2395	40	3.08
	*i*-PrOH	289 (61,094); 375 (33,679)	411	36/2336	61	3.07
	MeCN	289 (54,697); 374 (33,631)	413	39/2525	66	3.08
	DMSO	292 (51,300); 379 (31,082)	419	40/2519	75	3.03
	H_2_O	292 (47,398); 377 (25,558)	452	75/4401	4.6	3.02
**11d**	THF	293 (57,719); 335 (45,520); 363 (35,876)	411; 433	48/4507	59	2.98
	*i*-PrOH	294 (50,600); 334 (43,300); 363(31,200)	410; 432	47/4454	34	3.06
	MeCN	292 (48,179); 335 (41,047); 363 (31,120)	413; 433	50/4322	51	3.07
	DMSO	296 (47,815); 339 (39,355); 368 (29,565)	418; 440	50/4322	73	3.01
	H_2_O	297 (43,381); 340 (36,844); 370 (26,996)	425; 448	55/4793	4.4	3.06
**11g**	THF	308 (72,776); 346 (63,363)	457	111/6974	39	2.95
	*i*-PrOH	308 (77,302); 346 (65,169)	496	150/8751	36	2.90
	MeCN	308 (76,261); 349 (65,170)	543	194/10,237	11	2.94
	DMSO	313 (70,499); 353 (62,308)	550	197/12,800	0.48	2.93
	H_2_O	312 (63,524); 346 (50,274)	506	160/9175	1.6	2.94

^1^ Eg^opt^ was calculated on the direct application of the Tauc plot.

## Data Availability

Dataset available on request from the authors.

## Supplementary Materials

The following supporting information can be downloaded at: https://www.mdpi.com/article/10.3390/molecules30234508/s1: Figure S1: UV-Vis absorption (solid line) and photoluminescence (PL, dash line) emission spectra of compound **10f** in THF and MeCN (C = 10^−5^ M); Figure S2: Plots of TCSPC for **10c** in various solvents; Figure S3: Plots of TCSPC for **10d** in various solvents; Figure S4: Plots of TCSPC for **10g** in various solvents; Table S1: Radiative and nonradiative decay rates of **10c**, **10d** and **10g** in various solvents; Tauc plots; Copies of NMR ^1^H, ^13^C.
