# Supplementary material for: Triazole-Based Functionalized Olygo(Arylene Ethynylene)s—Synthesis and Properties
Source: Molecules. 2025 Nov 22;30(23):4508. doi: 10.3390/molecules30234508 (PMC12692853; doi:10.3390/molecules30234508)
Supplement: Supplementary file 1 [file molecules-30-04508-s001.zip › molecules-3947886-supplementary.pdf]

SUPPLEMENTARY INFORMATION

Triazole-Based Functionalized Olygo(Arylene Ethynylene)s—Synthesis and Properties

Anastasia I. Govdi [1], Vasiliy V. Menchikov [1], Ilya E. Kolesnikov [2] and Irina A. Balova [1,*]

[1] Institute of Chemistry, Saint Petersburg State University (SPbU), Universitetskaya nab. 7/9, 199034 Saint Petersburg, Russia; a.govdi@spbu.ru (A.I.G.); st106275@student.spbu.ru (V.V.M.)
[2] Center for Optical and Laser Materials Research, Saint Petersburg State University, Ulianovskaya 5, 198504 Saint Petersburg, Russia; ilya.kolesnikov@spbu.ru
* Correspondence: i.balova@spbu.ru; Tel.: +7-812-4286733

Table of Contents

1. Absorption and emission spectra of **10f**……………………………………………………..S3
2. Luminescence kinetic curves for **10c**, **10d** and **10g**…………………………………..S3
3. Rates of radiative (k_r) and nonradiative (k_{nr}) decay…………………………………………...S5
4. Tauc plots…………………………………………………………………………………………………..S6
5. Copies of NMR ^{1}H, ^{13}C…………………………………………………………………………………S17

1. Absorption and emission spectra of 10f

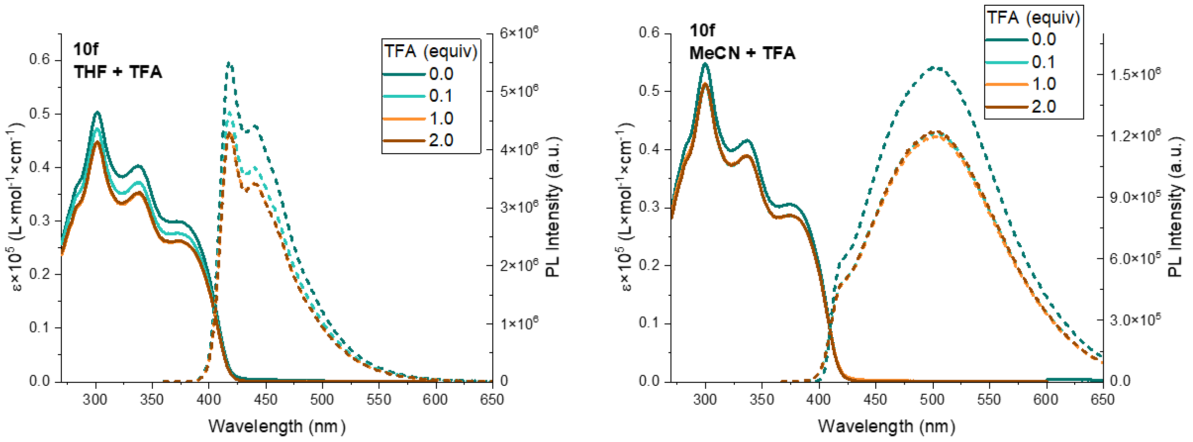

Figure S1. UV-Vis absorption (solid line) and photoluminescence (PL, dash line) emission spectra of compound **10f** in THF and MeCN (C = 10^{-5} M).

2. Luminescence kinetic curves for 10c, 10d and 10g

Luminescence kinetic curves for **10c**, **10d** and **10g** in various solvents have been presented in below along the corresponding data fitting as recommended. These samples were selected as illustrative examples. The majority of experimental decay curves were fitted by single exponential function. However, several samples (especially in water and DMSO) required a bi-exponential fitting.

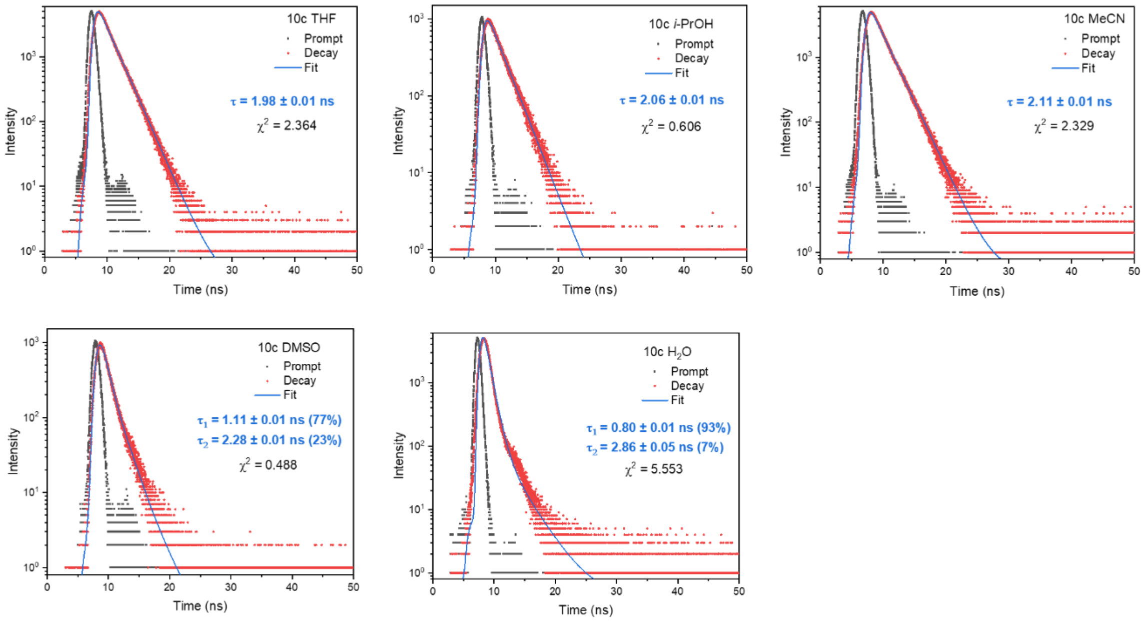

Figure S2. Plots of TCSPC for **10c** in various solvents

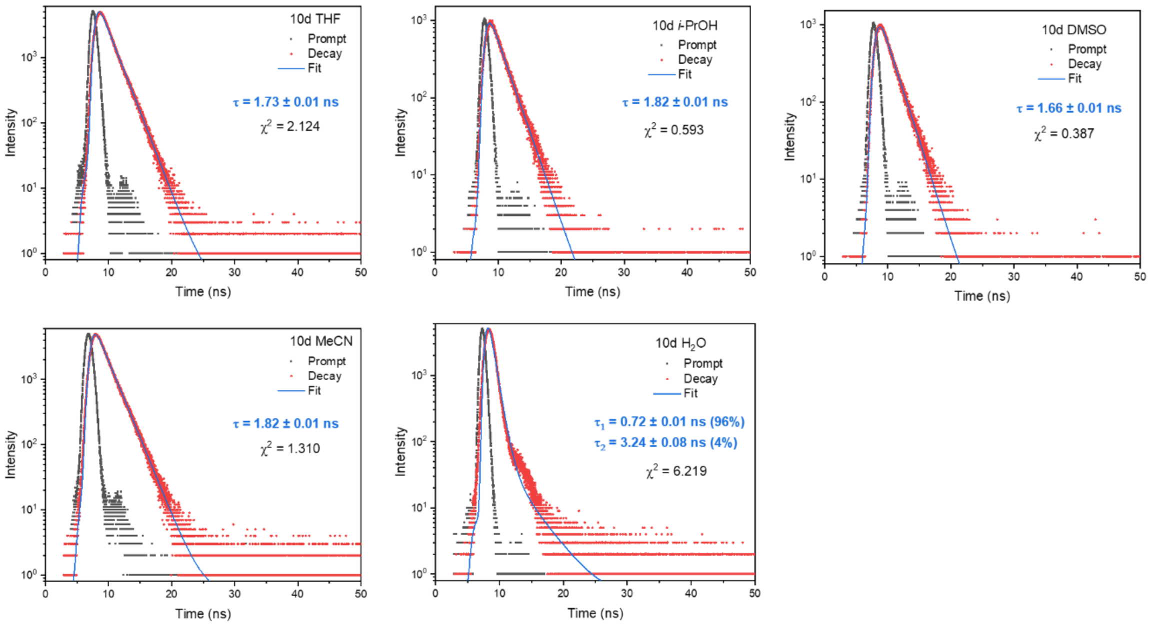

Figure S3. Plots of TCSPC for **10d** in various solvents

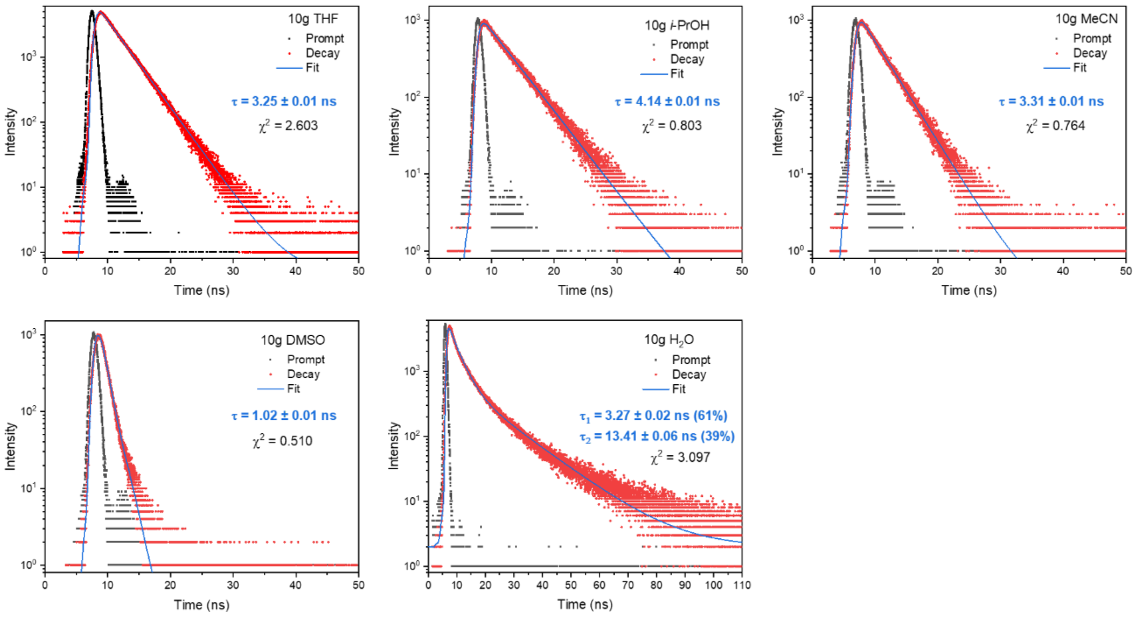

Figure S4. Plots of TCSPC for **10g** in various solvents

3. Rates of radiative (k_r) and nonradiative (k_{nr}) decay

The rates of radiative (k_r) and nonradiative (k_{nr}) decay for compounds 10c, 10d, and 10g were calculated for a number of solvents using the following formulae:

$k_r = QY/\tau$

$k_{nr} = (1-QY)/\tau$

The calculation data is presented in Table S1.

Table S1. Radiative and nonradiative decay rates of **10c, 10d and 10g** in various solvents

**Comp.**	**Solvent**	**k_r, s^{-1}**	**k_{nr}, s^{-1}**		**Solvent**	**k_r, s^{-1}**	**k_{nr}, s^{-1}**
**10c**	THF	4.4E8	6.1E7	**10d**	THF	3.6E8	2.1E8
	i-PrOH	3.2E8	1.7E8		*i*-PrOH	2.7E8	2.7E8
	MeCN	3.6E8	1.2E8		MeCN	4.3E8	1.2E8
	DMSO	6.0E8	1.2E8		DMSO	3.9E8	2.1E8
	Water	1.6E8	8.9E8		Water	1.6E8	1.1E9
**10g**	THF	1.7E8	1.4E8				
	i-PrOH	7.2E7	1.7E8				
	MeCN	3.6E7	2.7E8				
	DMSO	6.9E7	9.1E8				
	Water	2.2E7	1.2E8				

4. Tauc plots

The band gap energy was determined using the Tauc method. The fundamental equation of this method describes the relationship between the absorption coefficient and the photon energy.

$$(A \times h \times \nu)^2 = K \times (h \times \nu - E_g)$$

Where:

- **A** is the absorbance (optical density)
- **h** is Planck's constant
- **v** is the photon frequency
- **K** is a constant
- **Eg** is the band gap energy

First, the absorption spectrum was transformed: the wavelength axis was converted to a photon energy (h × v) axis, and the absorbance axis was converted to the square of the product of absorbance and photon energy, $(A \times h \times v)^2$. Subsequently, the linear portion of the dependence at the absorption edge was identified on the spectrum. This region was fitted with a linear equation (y = a × (hv) + b), and the point where this line intersects the abscissa axis (x-axis) was determined using the equation Eg = -b/a. The band gap energy was thus determined.

Compound 10a

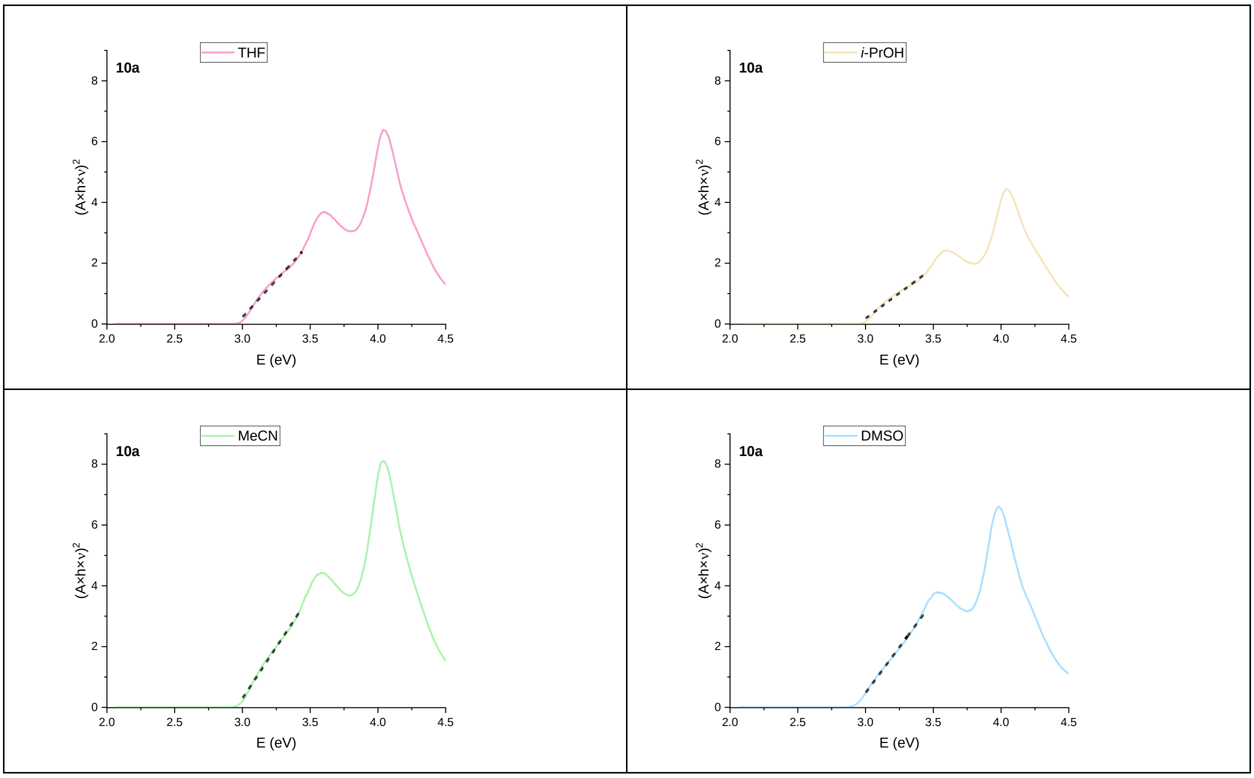

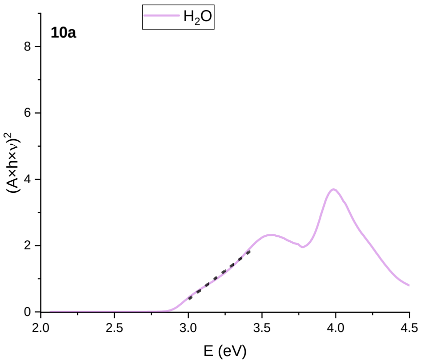

Solvent	THF	*i*-PrOH	MeCN	DMSO	H_2O
b	-14.45 ± 0.18	-9.84 ± 0.14	-19.98 ± 0.14	-17.62 ± 0.12	-10.06 ± 0.11
a	4.89 ± 0.06	3.34 ± 0.04	6.76 ± 0.04	6.03 ± 0.04	3.48 ± 0.04
r^2	0.98686	0.98214	0.99577	0.99613	0.98917
E, eV	2.96	2.95	2.96	2.92	2.89

Compound 10c

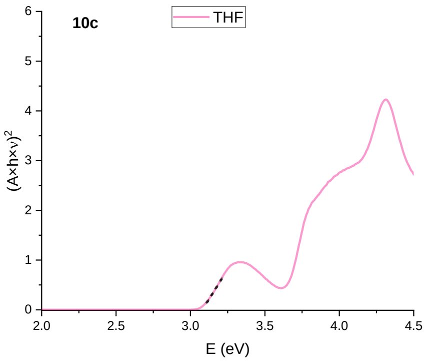

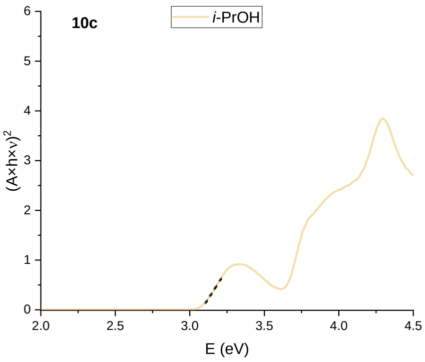

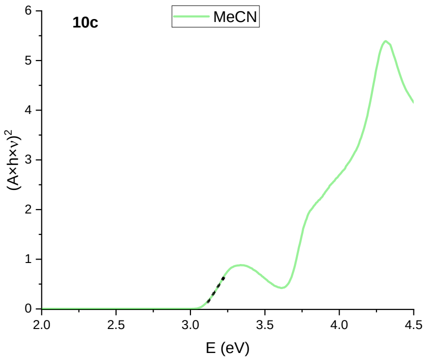

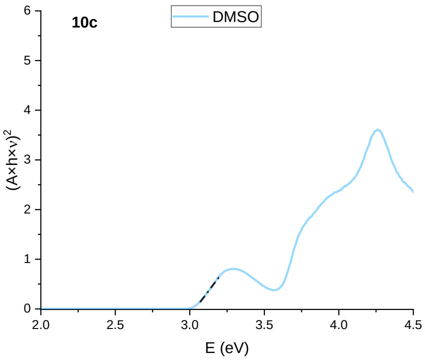

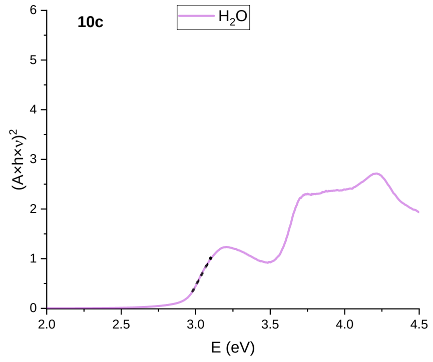

Solvent	THF	*i*-PrOH	MeCN	DMSO	H_2O
b	-14.69 ± 0.16	-14.37 ± 0.18	-14.37 ± 0.18	-12.14 ± 0.07	-16.07 ± 0.16
a	4.77 ± 0.05	4.67 ± 0.06	4.65 ± 0.06	4.00 ± 0.02	5.51 ± 0.05
r^2	0.99717	0.99593	0.99598	0.99895	0.997
E, eV	3.08	3.08	3.09	3.04	2.92

Compound 10d

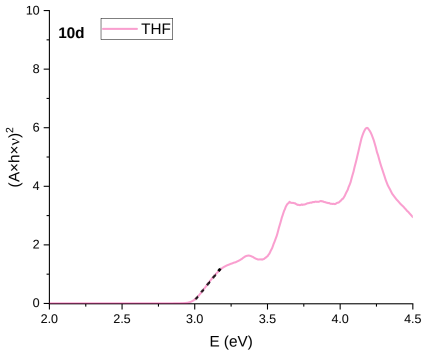

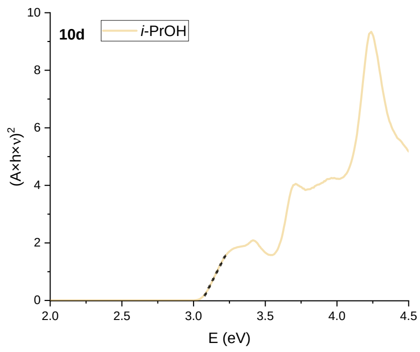

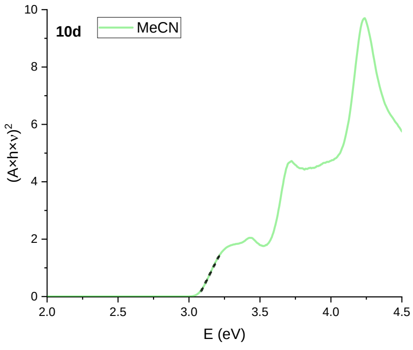

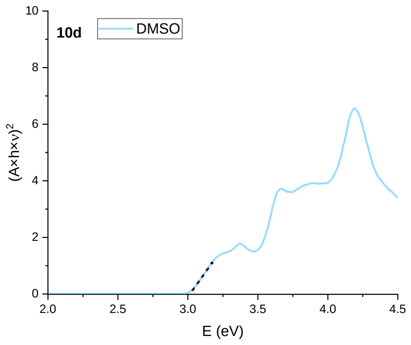

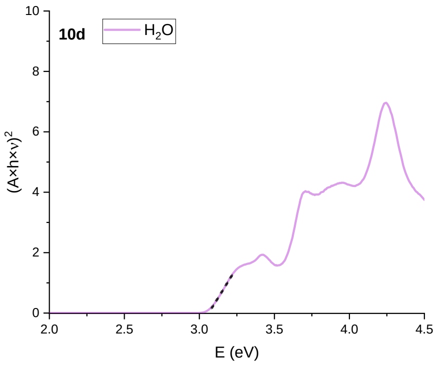

Solvent	THF	*i*-PrOH	MeCN	DMSO	H_2O
b	-18.25 ± 0.10	-29.7 ± 0.2	-29.8 ± 0.2	-21.06 ± 0.16	-24.59 ± 0.19
a	6.12 ± 0.03	9.71 ± 0.06	9.73 ± 0.07	6.99 ± 0.05	8.03 ± 0.06
r^2	0.99873	0.99852	0.99806	0.99797	0.99794
E, eV	2.98	3.06	3.06	3.01	3.06

Compound 10e

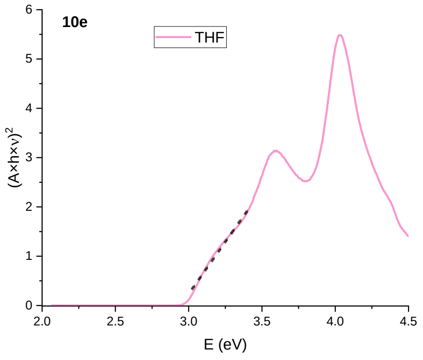

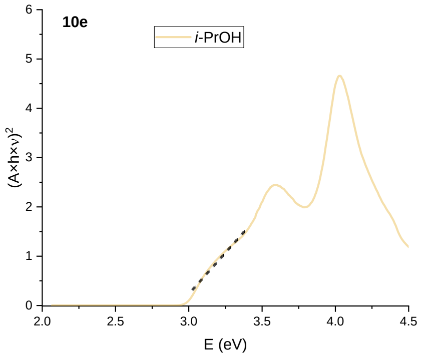

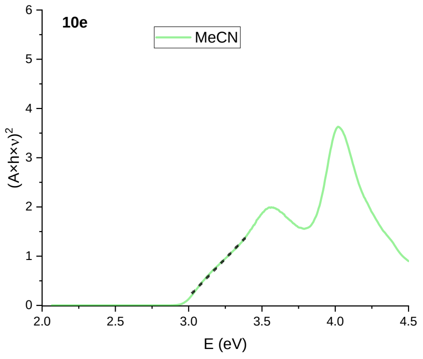

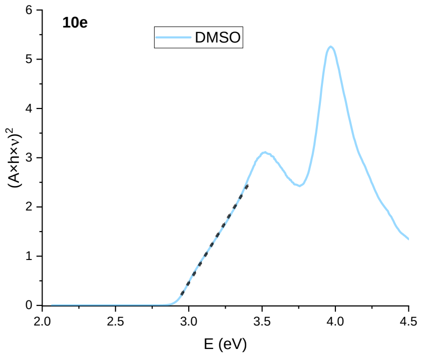

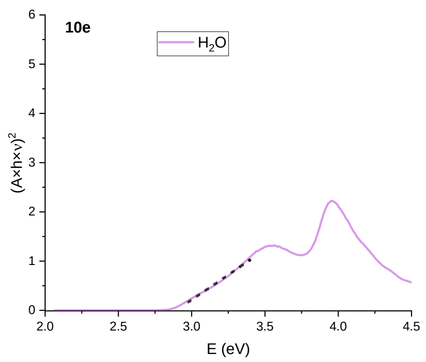

Solvent	THF	*i*-PrOH	MeCN	DMSO	H_2O
b	-12.40 ± 0.15	-9.78 ± 0.15	-9.11 ± 0.05	-14.31 ± 0.06	-5.89 ± 0.06
a	4.21 ± 0.05	3.34 ± 0.05	3.097 ± 0.016	4.92 ± 0.02	2.04 ± 0.02
r^2	0.98837	0.98349	0.99771	0.99831	0.99029
E, eV	2.95	2.93	2.94	2.91	2.89

Compound 10f

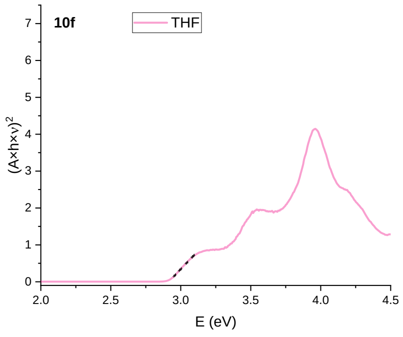

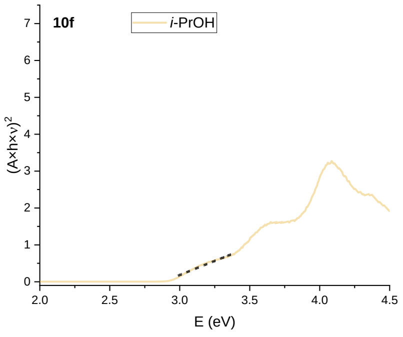

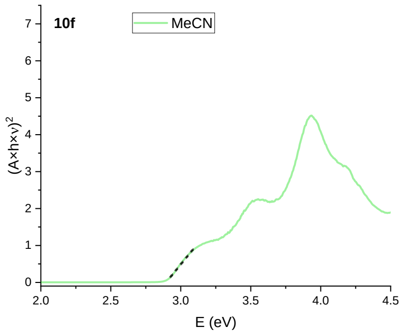

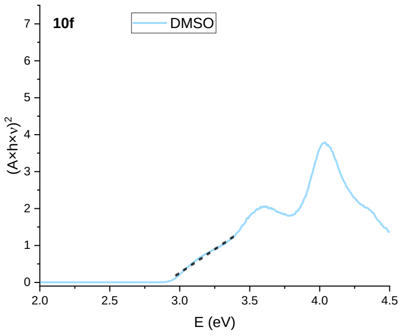

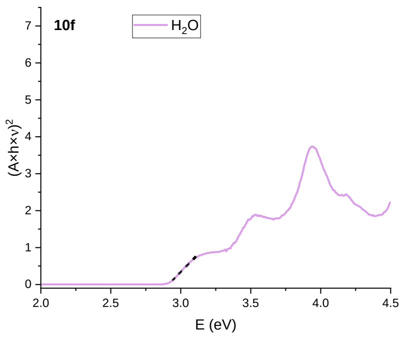

Solvent	**THF**	***i*-PrOH**	**MeCN**	**DMSO**	**H_2O**
b	-11.61 ± 0.08	-4.39 ± 0.07	-13.41 ± 0.15	-7.40 ± 0.08	-11.22 ± 0.11
a	3.98 ± 0.03	1.52 ± 0.02	4.63 ± 0.05	2.55 ± 0.03	3.85 ± 0.03
r^2	0.99846	0.97927	0.99502	0.98969	0.9964
E, eV	2.92	2.89	2.90	2.90	2.91

Compound 10g

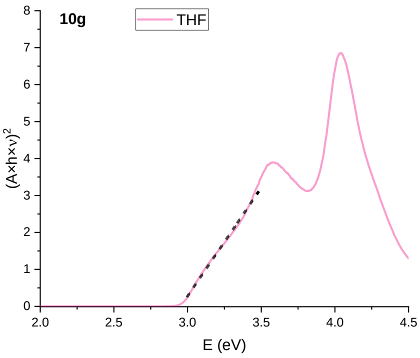

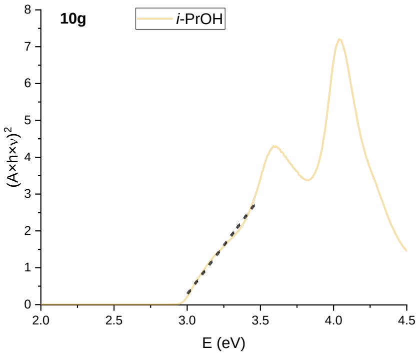

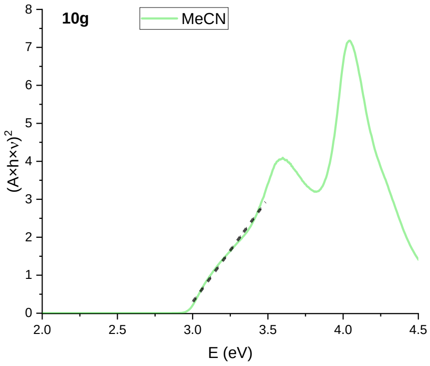

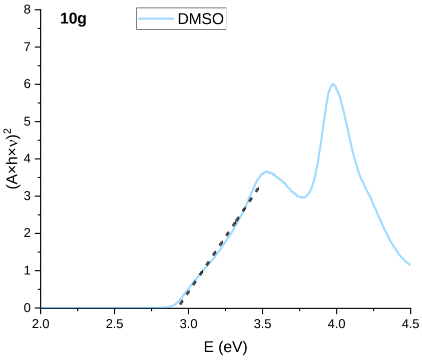

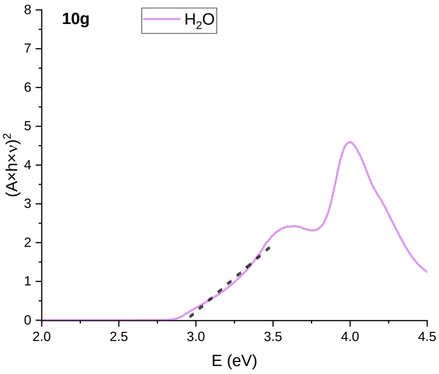

Solvent	THF	*i*-PrOH	MeCN	DMSO	H_2O
b	-17.31 ± 0.14	-15.6 ± 0.2	-16.04 ± 0.17	-17.3 ± 0.2	-10.17 ± 0.17
a	5.86 ± 0.04	5.29 ± 0.06	5.44 ± 0.05	5.90 ± 0.06	3.47 ± 0.05
r^2	0.99697	0.99272	0.99477	0.99297	0.98495
E, eV	2.95	2.95	2.95	2.93	2.93

Compound 11a

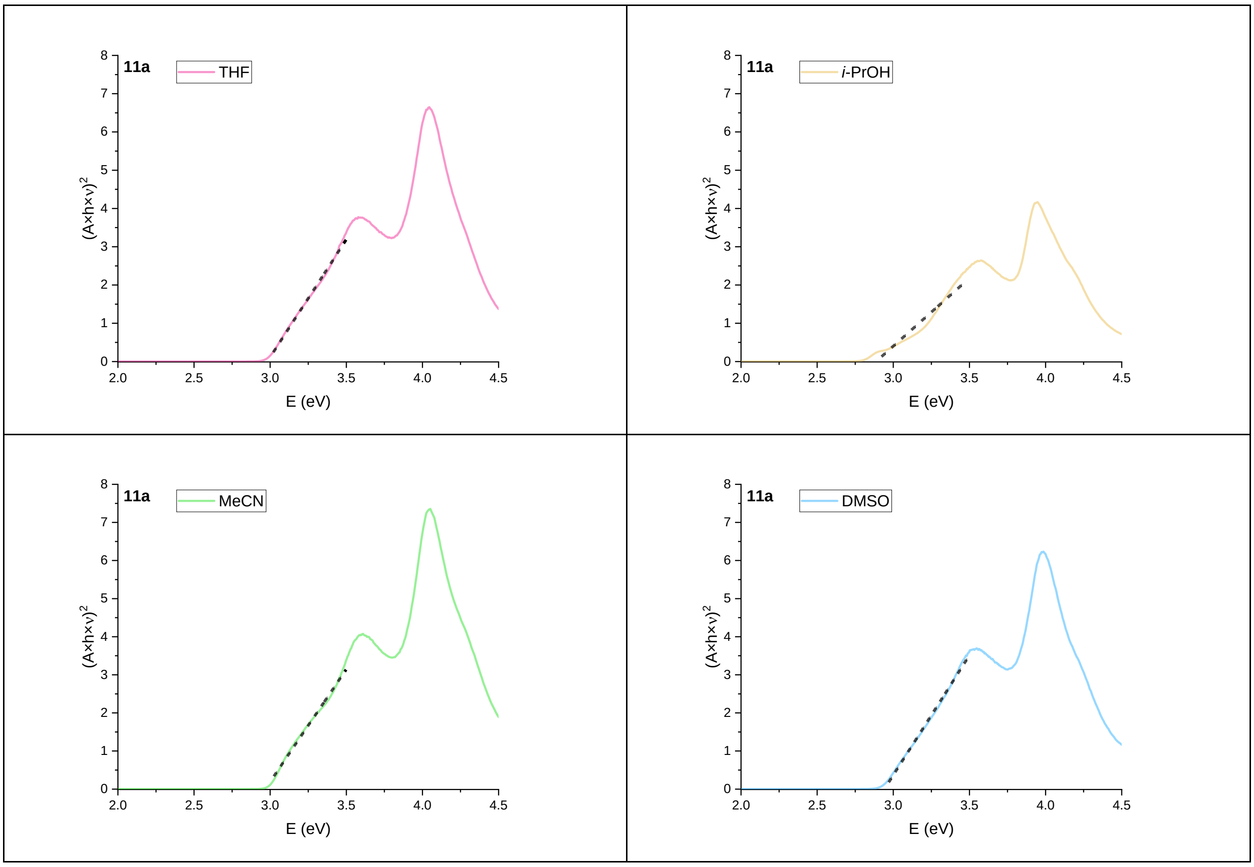

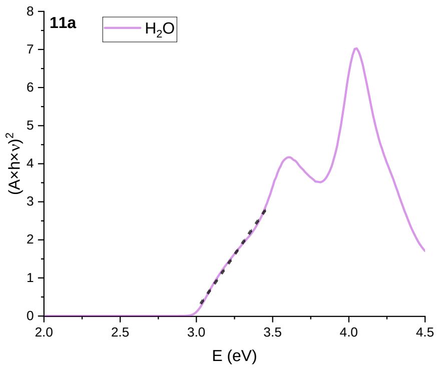

Solvent	THF	*i*-PrOH	MeCN	DMSO	H_2O
b	-18.34 ± 0.13	-18.34 ± 0.13	-10.3 ± 0.2	-17.50 ± 0.16	-17.50 ± 0.16
a	6.15 ± 0.04	6.15 ± 0.04	3.55 ± 0.08	5.90 ± 0.05	5.90 ± 0.05
r^2	0.99562	0.93578	0.99261	0.99261	0.99587
E, eV	2.98	2.98	2.90	2.97	2.97

Compound 11c

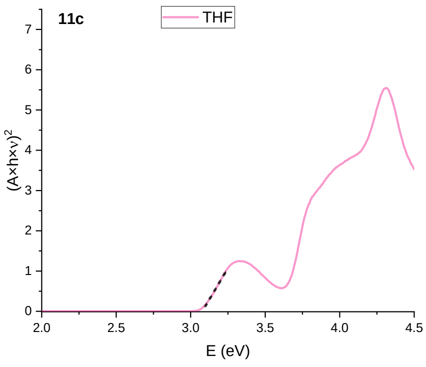

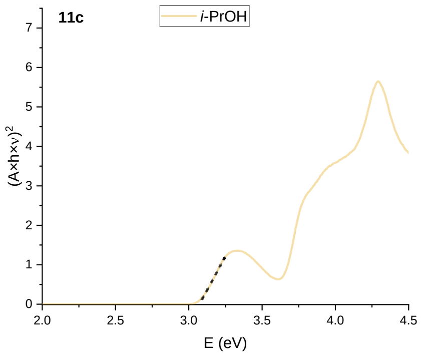

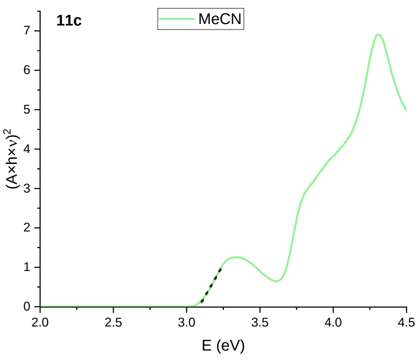

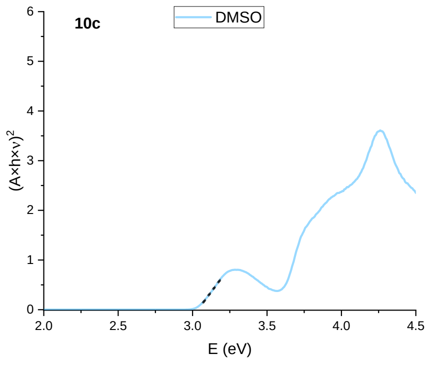

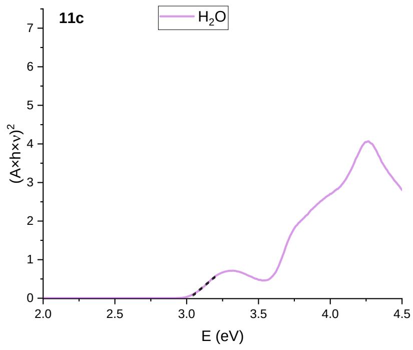

Solvent	THF	*i*-PrOH	MeCN	DMSO	H_2O
b	-19.17 ± 0.16	-21.0 ± 0.2	-19.9 ± 0.2	-15.27 ± 0.11	-9.42 ± 0.10
a	6.23 ± 0.05	6.83 ± 0.06	6.46 ± 0.08	5.04 ± 0.03	3.12 ± 0.03
r^2	0.99761	0.9966	0.99528	0.99811	0.99572
E, eV	3.08	3.07	3.08	3.03	3.02

Compound 11d

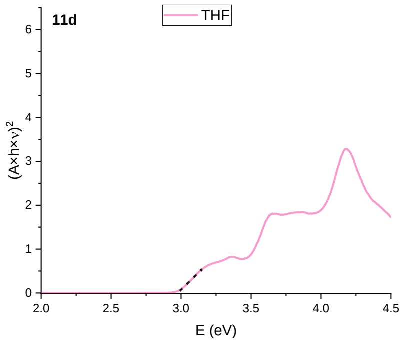

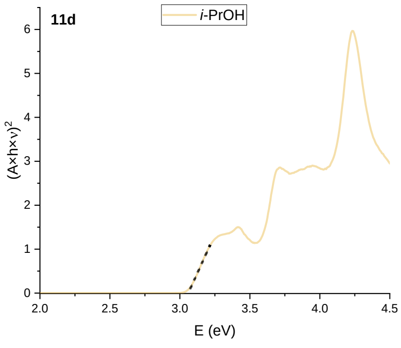

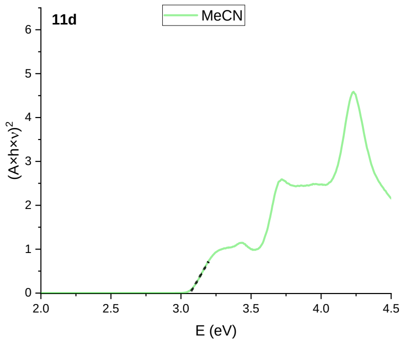

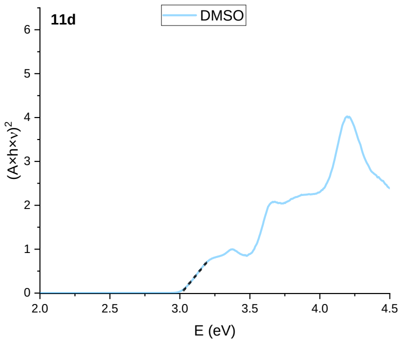

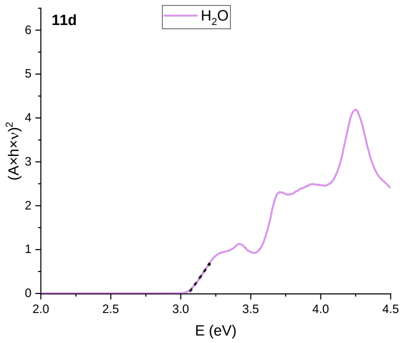

Solvent	THF	*i*-PrOH	MeCN	DMSO	H_2O
b	-9.29 ± 0.06	-21.44 ± 0.15	-16.70 ± 0.20	-11.66 ± 0.08	-13.76 ± 0.17
a	3.12 ± 0.02	7.01 ± 0.05	5.45 ± 0.06	3.87 ± 0.03	4.50 ± 0.05
r^2	0.99822	0.9984	0.99587	0.9982	0.99515
E, eV	2.98	3.06	3.06	3.01	3.06

Compound 11g

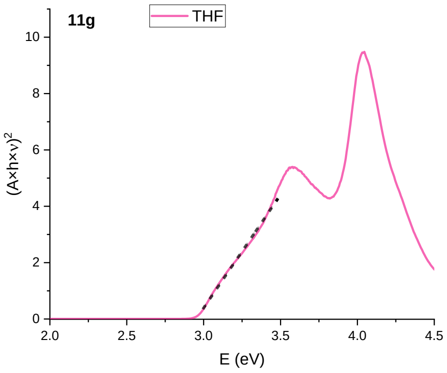

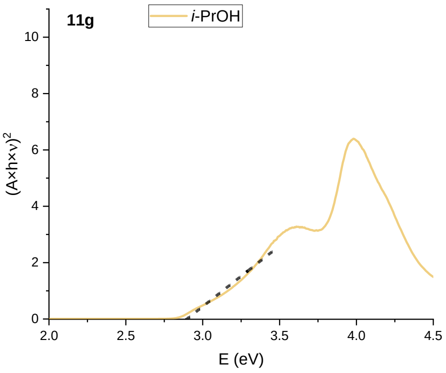

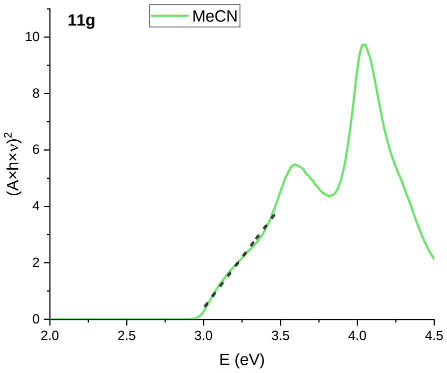

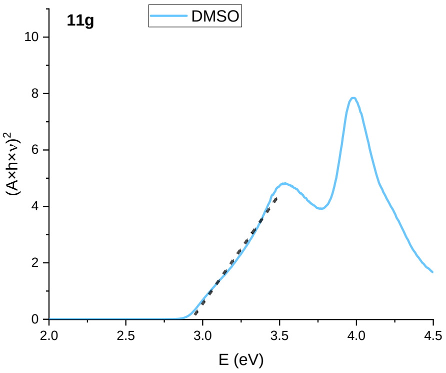

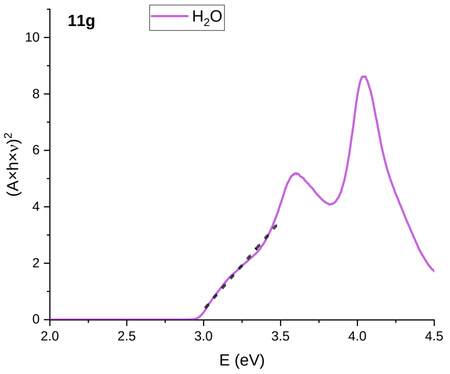

Solvent	THF	*i*-PrOH	MeCN	DMSO	H_2O
b	-23.7 ± 0.2	-12.5 ± 0.2	-21.2 ± 0.2	-22.8 ± 0.3	-18.6 ± 0.2
a	8.04 ± 0.06	4.31 ± 0.07	7.19 ± 0.07	7.77 ± 0.08	6.32 ± 0.08
r^2	0.99292	0.96723	0.98845	0.9853	0.98435
E, eV	2.95	2.90	2.95	2.93	2.94
